# *Arabidopsis* TPXL Proteins Mediate a Selective Aurora Kinase–Microtubule Association During Cell Division

**DOI:** 10.3390/ijms27187980

**Published:** 2026-09-08

**Authors:** Shilin Cao, Yuying Chen, Zongkuan Weng, Haiyun Ren, Pingzhou Du

**Affiliations:** 1 Center for Biological Science and Technology, Faculty of Arts and Sciences, Beijing Normal University, Zhuhai 519087, China; 2 Instrumentation and Service Center for Science and Technology, Beijing Normal University, Zhuhai 519087, China

**Keywords:** microtubule organization, microtubule-associated proteins, TPX2-like proteins, Aurora kinase, plant cell division, AlphaFold3

## Abstract

Microtubule organization during cell division requires coordinated regulation by microtubule-associated proteins and protein kinase signaling pathways. In animals, TPX2 (Targeting Protein for Xklp2) regulates spindle assembly by binding to and activating Aurora A kinase. In *Arabidopsis*, individual TPX2-like (TPXL) proteins have been implicated in Aurora-associated functions, but whether the TPXL family members share common microtubule-associated properties and how TPXL-Aurora interactions differ across the family remain incompletely understood. Here, we investigated the structural and cellular relationships among the eight *Arabidopsis* TPX2-like proteins (TPXL1–TPXL8), microtubules, and Aurora kinases. AlphaFold3-based structural prediction indicated that specific interaction sites between TPXL proteins and tubulins were revealed at the three-dimensional structure and atomic level, suggesting that all TPXL proteins contain a conserved TPX2 domain contributing to their predicted tubulin association. Transient expression analyses showed that TPXL1–TPXL8 localized to microtubule arrays during both interphase and cell division. Although microtubule-associated domain is predicted to be conserved across the family, individual TPXL members exhibit distinct localization patterns on spindles and phragmoplasts, suggesting potential functional specialization. Further structural prediction and experimental validation revealed that only TPXL2, TPXL3, TPXL4, and TPXL8 interacted with AUR1 (Aurora 1) and AUR2, whereas no detectable interactions were observed for the other TPXL members or for any TPXL protein with AUR3. Structural prediction of TPXL–AUR1–tubulin complexes were consistent with a possible arrangement in which TPXL proteins contact both AUR1 and tubulin. Together, our findings suggest that *Arabidopsis* TPXL proteins have broad microtubule association and are coupled with selective Aurora kinase interaction to coordinate microtubule organization, providing a framework for future functional analysis of TPXL–Aurora–microtubule association during plant cell division.

## 1. Introduction

Microtubules are highly dynamic tubulin polymers that play essential roles in a wide range of cellular processes including intracellular transport, cell polarity establishment, and cell division. During mitosis and meiosis, microtubules undergo extensive reorganization to generate specialized arrays, including the spindle that mediates chromosome segregation and the phragmoplast that directs cell plate formation in plant cytokinesis [1,2,3]. The formation and function of these microtubule arrays require precise regulation by microtubule-associated proteins (MAPs) and protein kinases that coordinate microtubule dynamics with cell cycle progression [4,5,6]. Among these regulatory factors, Aurora kinases are highly conserved serine/threonine protein kinases that function as key regulators of spindle assembly, chromosome segregation, and cytokinesis in eukaryotic organisms [7,8]. In *Arabidopsis*, three Aurora kinase genes, *AUR1*, *AUR2*, and *AUR3*, have been identified, and functional studies have revealed their important roles in cell division, chromosome segregation, and reproductive development [9].

In animal cells, the spatial regulation of Aurora kinase activity is tightly controlled by the spindle assembly factor TPX2 (Targeting Protein for Xklp2). TPX2 is a conserved microtubule-associated protein that contains a C-terminal TPX2 domain responsible for binding microtubules and an Aurora-binding region that interacts with Aurora A kinase [10,11,12]. During mitosis, TPX2 recruits Aurora A to spindle microtubules and promotes its activation, thereby restricting Aurora A activity to specific spindle regions required for bipolar spindle assembly and chromosome segregation. In addition, Aurora A phosphorylates TPX2, suggesting that TPX2 and Aurora A form a coordinated regulatory module to control spindle organization [13]. The TPX2–Aurora A regulatory module represents an important mechanism linking protein kinase signaling with microtubule organization.

Although TPX2-like proteins have been identified in plants, how they function remains largely unclear. In *Arabidopsis*, eight TPX2-like proteins (TPXL1–TPXL8) containing conserved TPX2 domains have been identified [14]. Previous studies have suggested that several TPXL proteins are involved in plant development and reproductive processes. For example, mutants with suppressed TPXL3 expression exhibited severe developmental and cell division defects [15,16]. However, whether the conserved TPX2-like microtubule association and Aurora kinase interaction capacity are uniformly maintained across the entire TPXL family remains unclear. Specifically, it is unknown whether Aurora-binding capability is conserved among TPXL family members, and how TPXL proteins may coordinate Aurora kinase localization with microtubule organization.

The functional diversification of Aurora kinases in *Arabidopsis* further raises the possibility that different TPXL proteins may selectively regulate distinct Aurora-dependent processes. AUR1 and AUR2 have mainly been implicated in mitotic spindle organization and cytokinesis, whereas AUR3 has been associated with centromere-related functions and chromosome segregation through regulation of the centromeric histone variant CENH3 [9]. Whether TPXL proteins serve as common regulators of Aurora kinases or preferentially associate with specific Aurora members remains unknown.

In this study, we investigated the structural and cellular relationships among *Arabidopsis* TPXL proteins, microtubules, and Aurora kinases. Using AlphaFold3-based structural prediction, specific interaction sites between TPXL proteins and tubulins were revealed at the three-dimensional structure and atomic level, we found that all eight TPXL proteins had a conserved TPX2 domain-mediated association with tubulins, whereas TPXL proteins interacting with Aurora were selectively retained in a subset of TPXL members. Subcellular localization analyses implicated that TPXL1–TPXL8 associated with microtubule arrays during both interphase and cell division, while several members displayed distinct localization patterns on spindles and phragmoplasts. Furthermore, biochemical and cellular interaction assays identified TPXL2, TPXL3, TPXL4, and TPXL8 as Aurora-interacting proteins, and structural modeling predicted that these TPXL proteins connected Aurora kinase with tubulin. Together, our findings suggest that *Arabidopsis* TPXL proteins comprise the diversified TPX2-like family that may link Aurora kinase signaling with microtubule organization, providing a cue for future functional analysis during cell division.

## 2. Results

### 2.1. AlphaFold3 Predicts the Interactions Between TPXL Proteins and Tubulins

TPX2 family proteins are regarded as conserved microtubule-associated proteins that interact with microtubules and regulate spindle microtubule organization. To investigate whether *Arabidopsis* TPXL proteins retain the structural features required for microtubule association, we performed AlphaFold3-based structural prediction to analyze the potential interaction between TPXL proteins and α/β-tubulin heterodimers [17].

*Arabidopsis* contains multiple α- and β-tubulin isoforms (TUA/TUB) that exhibit distinct expression patterns. To select appropriate tubulin components for structural modeling, two representative α/β-tubulin combinations were analyzed, including TUA5/TUB6 and TUA2/TUB4. TUA5 and TUB6 are widely expressed representative tubulin isoforms in *Arabidopsis* and are frequently used as representative tubulin components in structural and functional studies [18]. In contrast, TUA2 and TUB4 show relatively high expression levels in floral tissues [19], and previous studies have suggested that some TPXL family members are involved in cell division and that the disruption of TPXL function affects gametophyte development [15,16]. Therefore, TUA2–TUB4 was included as a biologically relevant tubulin pair associated with reproductive tissues.

**Figure 1:**
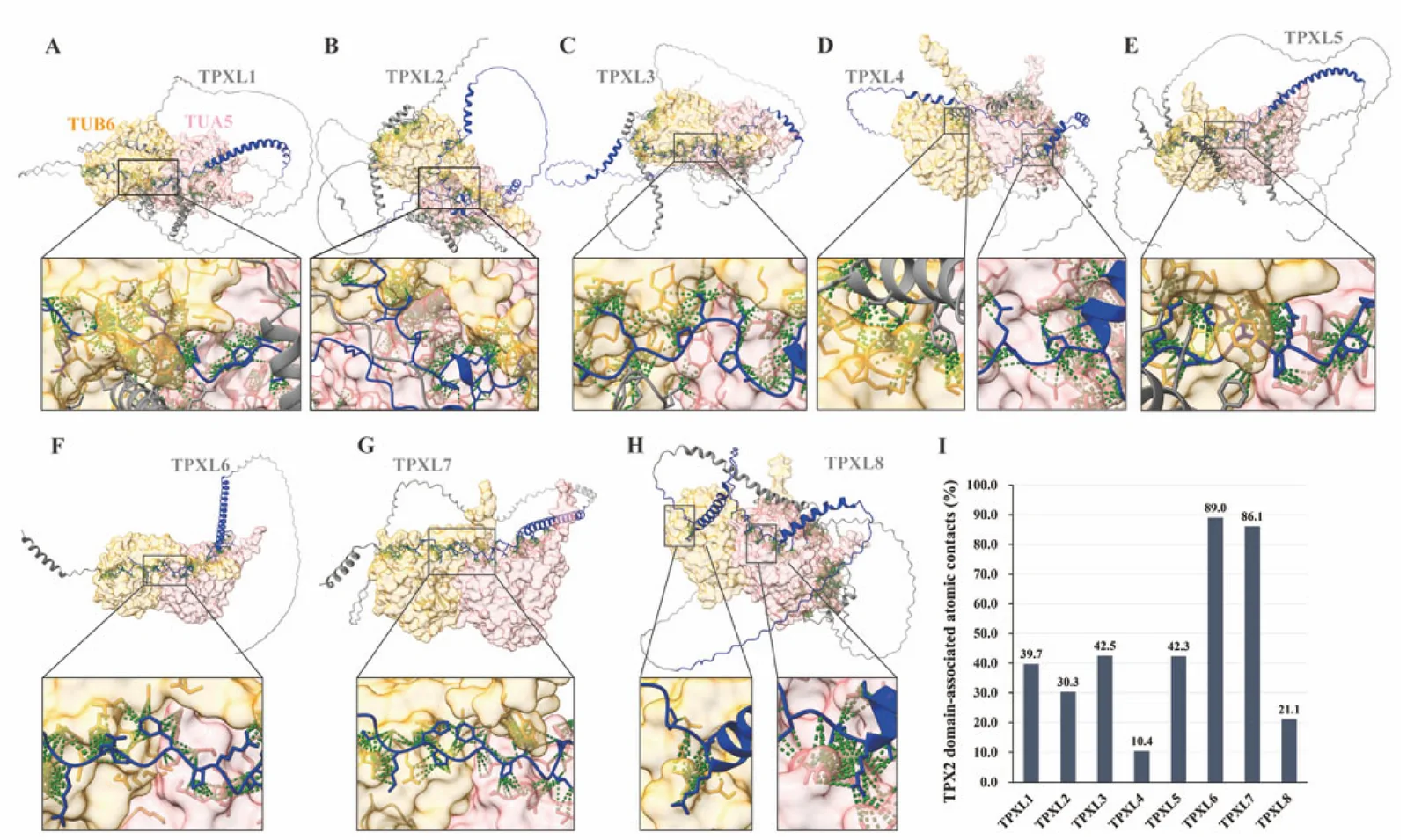
AlphaFold3 predicts conserved TPX2 domain-associated tubulin contacts in *Arabidopsis* TPXL proteins. (**A**–**H**) Predicted structural models of TPXL1–TPXL8 proteins in complex with the tubulin heterodimer generated using AlphaFold3. TPXL proteins are shown in gray, with the conserved TPX2 domains highlighted in blue. The α- and β-tubulin subunits TUA5 and TUB6 are shown in pink and orange-yellow, respectively. Predicted atomic contacts between TPXL proteins and tubulin with interatomic distances shorter than 4.5 Å are indicated by green dashed lines. (**I**) Quantification of the contribution of the conserved TPX2 domain to the predicted TPXL–tubulin interfaces. The percentage of TPX2 domain-associated atomic contacts was calculated as the number of predicted atomic contacts within the annotated TPX2 domain divided by the total number of predicted intermolecular atomic contacts between each TPXL protein and tubulin. Data are shown as the percentage contribution of the TPX2 domain for TPXL1–TPXL8.

AlphaFold3 prediction showed that all TPXL proteins were capable of forming complexes with both tubulin combinations. Importantly, the predicted interaction confidence scores of TPXL proteins with TUA5/TUB6 and TUA2/TUB4 were comparable, with similar interface predicted template modeling (ipTM) and predicted template modeling (pTM) values (Figure S1A), indicating that the predicted TPXL–tubulin association was not dependent on a specific tubulin isoform combination. Based on the broad expression pattern and extensive application of TUA5 and TUB6 as representative tubulin subunits, the TUA5/TUB6 complex was selected for subsequent structural analysis. The predicted TPXL/TUA5/TUB6 complexes revealed that all eight TPXL proteins could associate with the tubulin heterodimer (Figure 1A–H). The predicted interaction confidence scores showed relatively high reliability, with ipTM and pTM values ranging from 0.6 to 0.8 (Figure S1A). Furthermore, the predicted aligned error (PAE) plots showed lower uncertainty at the predicted interaction interfaces, supporting the structural compatibility between TPXL proteins and tubulin heterodimers (Figure S1B,C).

To further define the structural basis underlying TPXL–tubulin association, we identified predicted atomic contacts between TPXL proteins and tubulin heterodimers using an interatomic distance cutoff of 4.5 Å. The contacting atoms were mapped to the corresponding TPXL amino acid positions and classified according to the TPX2 domain annotation. Both TPX2 domain and non-TPX2 domain regions contributed to predicted tubulin contacts in all TPXL members, with TPX2 domain-associated contacts highlighted in dark blue and non-TPX2 domain-associated contacts shown in light blue (Figure S2A–D,I–L). The conserved TPX2 domains of all eight TPXL proteins showed direct spatial proximity to tubulin heterodimers, supporting a potential contribution of this domain to TPXL–tubulin association. Although the contribution of the TPX2 domain varied among TPXL members, TPX2 domain-derived residues accounted for 39.7%, 30.3%, 42.5%, 10.4%, 42.3%, 89.0%, 86.1%, and 21.1% of the total predicted tubulin-contacting residues in TPXL1–TPXL8, respectively (Figure 1I and Figure S2). Among these proteins, TPXL6 and TPXL7 exhibited the highest proportion of TPX2 domain-mediated contacts, suggesting that their interactions with tubulin may rely more strongly on this conserved region. To evaluate the structural confidence of different regions within TPXL proteins, predicted local distance difference test (pLDDT) scores were analyzed. The TPX2 domains exhibited consistently high confidence values (>70), whereas most non-TPX2 domain regions displayed lower pLDDT values (approximately 20–50) (Figure S2E–H,M–P), suggesting that the TPX2 domain represents a well-folded conserved structural element, whereas other regions may exhibit greater flexibility.

Together, these structural analyses suggest that *Arabidopsis* TPXL proteins possess a conserved TPX2 domain contributing to mediating predicted tubulin association, providing structural support for their potential roles as microtubule-associated proteins.

### 2.2. TPXL Family Proteins Associate with Cortical Microtubules During Interphase and Show Distinct Mitotic Localization Patterns

Canonical TPX2 proteins are known to associate with microtubule arrays through their conserved TPX2 domain [20]. To determine whether *Arabidopsis* TPXL proteins retain the microtubule association property of the TPX2 family, we analyzed the subcellular localization patterns of TPXL1–TPXL8 proteins in living plant cells.

**Figure 2:**
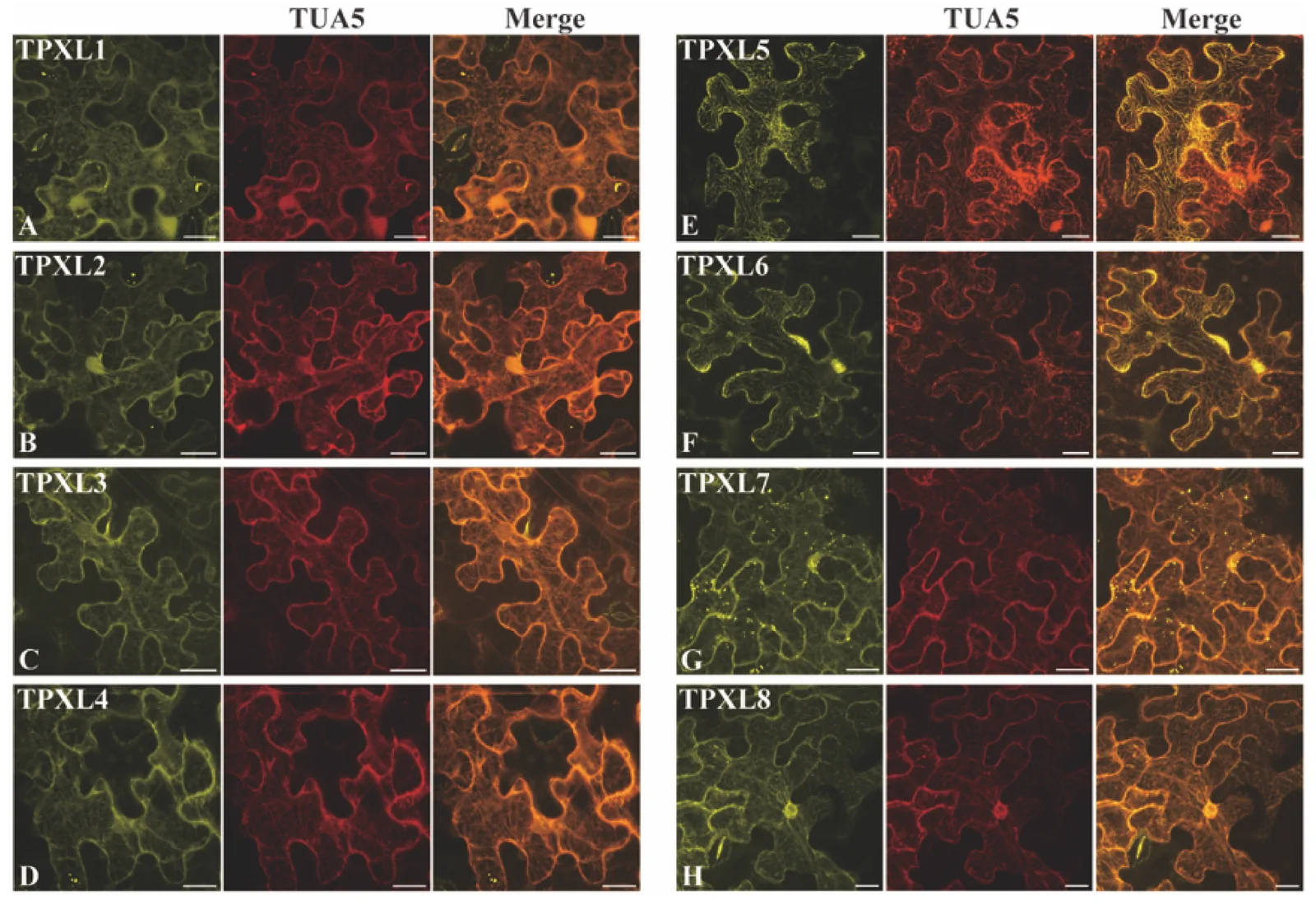
TPXL family proteins colocalize with cortical microtubules during interphase. (**A**–**H**) Subcellular localization of mCitrine-TPXL1–TPXL8 fusion proteins (yellow) in *Nicotiana benthamiana* leaf epidermal cells. TPXL proteins were expressed under the control of the *UBQ14* promoter. Cortical microtubules were visualized by the co-expression of tdTomato-TUA5 (red). MCitrine signals, tdTomato signals, and merged images are shown. All experiments were repeated three times, and at least 3 cells were analyzed each time. Scale bars, 20 μm.

The coding sequences of *TPXL1–TPXL8* were fused with *mCitrine* under the control of the constitutive *UBQ14* (*Ubiquitin 14*) promoter and transiently expressed in *Nicotiana benthamiana* leaf epidermal cells using an Agrobacterium-mediated transformation system. Microtubule arrays were visualized by the co-expression of tdTomato-TUA5, and the co-localization patterns were examined using confocal laser scanning microscopy. During interphase, all eight mCitrine-TPXL fusion proteins exhibited filamentous localization patterns that overlapped extensively with cortical microtubule arrays (Figure 2), supporting an association of TPXL family members with microtubule structures in this transient-expression system.

**Figure 3:**
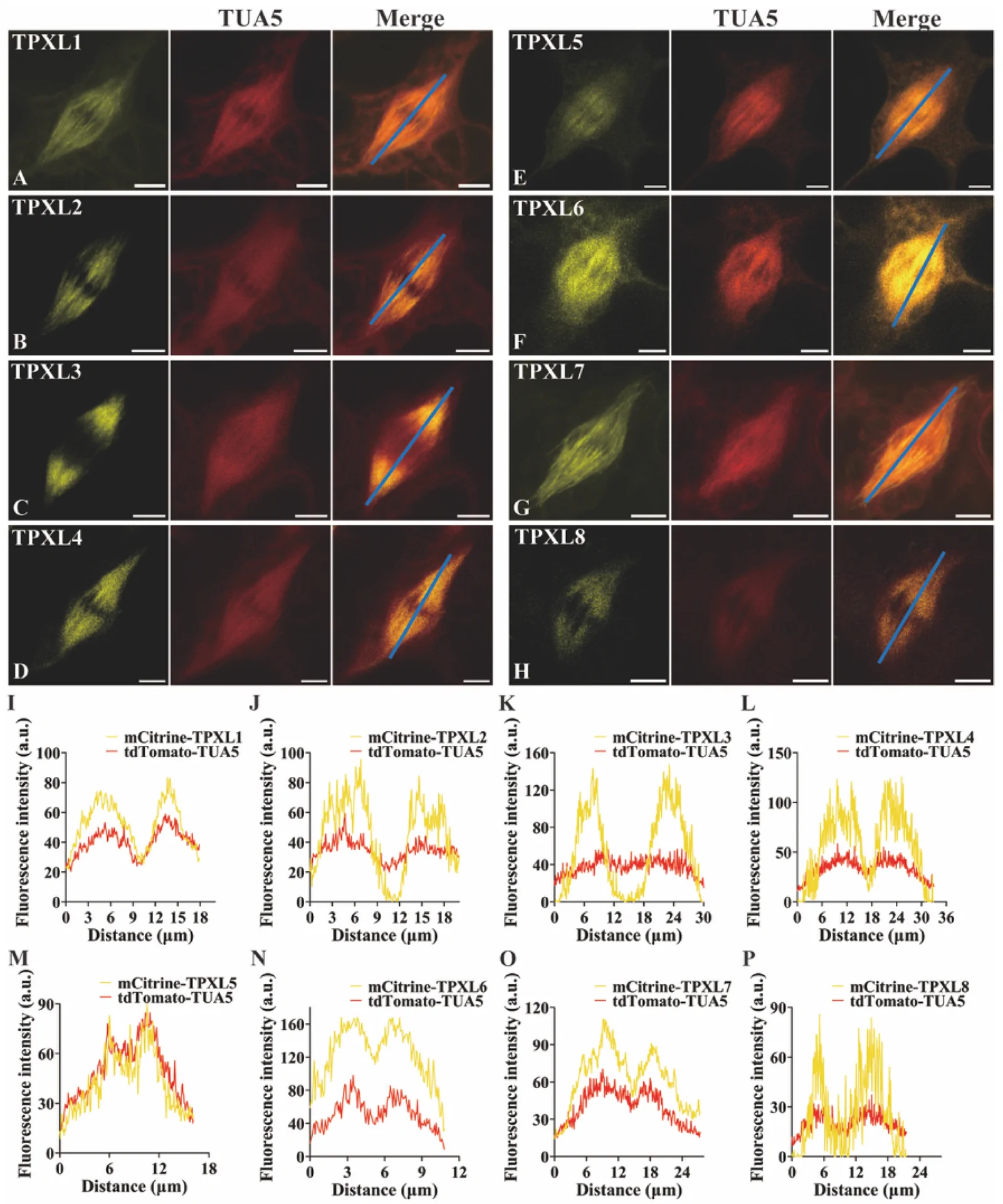
Differential localization of TPXL proteins on spindle microtubules during metaphase. (**A**–**H**) Localization of mCitrine-TPXL1–TPXL8 proteins (yellow) during metaphase in *Nicotiana benthamiana* leaf epidermal cells expressing AtCYCD3;1. Spindle microtubules were marked by tdTomato-TUA5 (red). Most TPXL proteins showed uniform spindle localization, whereas TPXL3 exhibited preferential accumulation at spindle poles. All experiments were repeated three times, and at least 3 cells were analyzed each time. Scale bars, 5 μm. (**I**–**P**) Fluorescence intensity profile analysis of TPXL proteins and microtubules. The blue lines indicate the regions selected for line-scan analysis. Relative fluorescence intensity profiles of mCitrine-TPXL proteins and tdTomato-TUA5 along the indicated axis were plotted to evaluate the spatial distribution of TPXL proteins on spindle microtubules.

**Figure 4:**
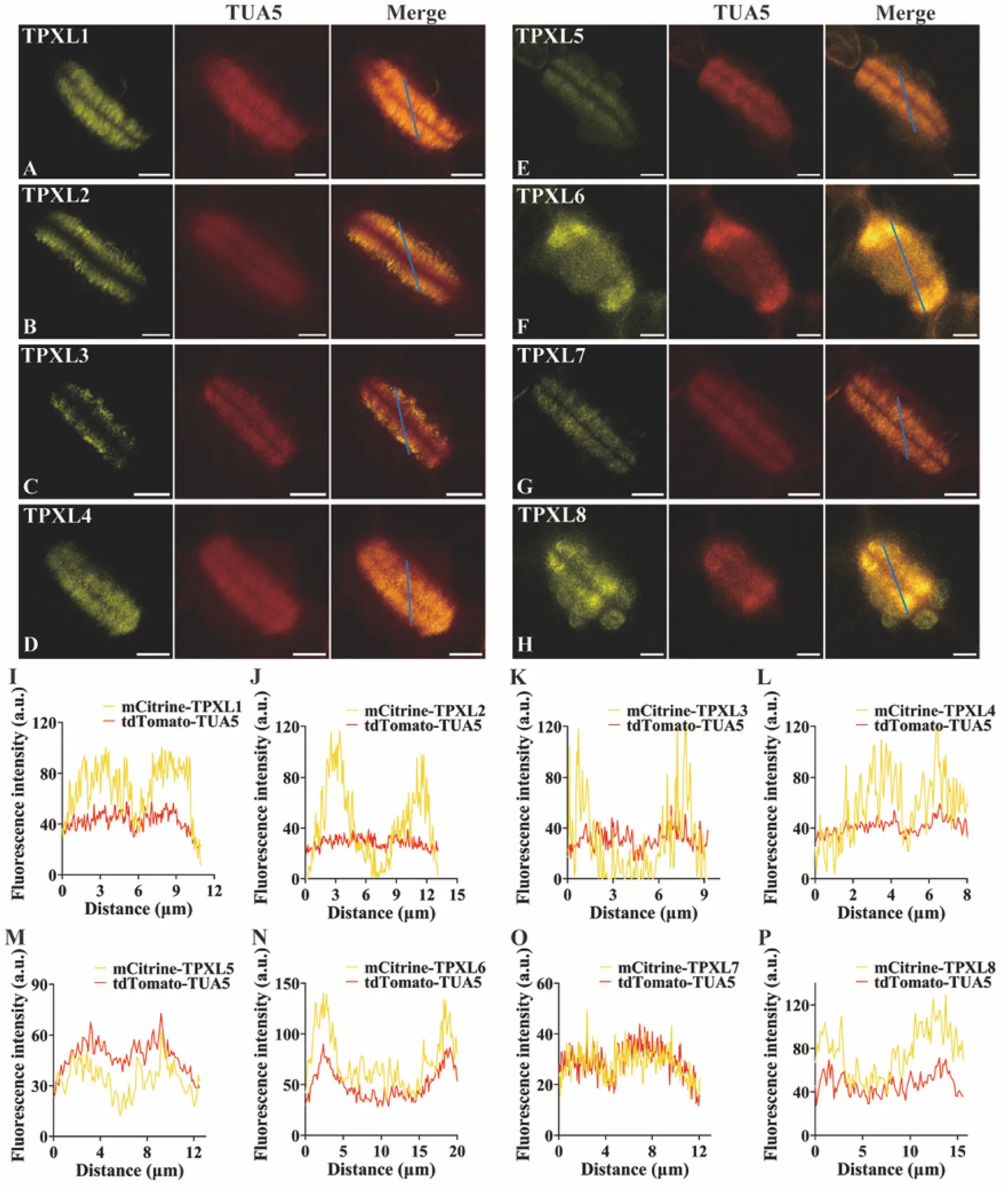
TPXL proteins localize to phragmoplast microtubules during cytokinesis. (**A**–**H**) Localization of mCitrine-TPXL1–TPXL8 proteins (yellow) during cytokinesis in AtCYCD3;1-induced dividing leaf epidermal cells in *Nicotiana benthamiana*. Phragmoplast microtubules were visualized by tdTomato-TUA5 (red). TPXL2 and TPXL3 showed preferential enrichment at or near the distal ends of the phragmoplast microtubules, whereas other TPXL proteins displayed a more uniform distribution. All experiments were repeated three times, and at least 3 cells were analyzed each time. Scale bars, 5 μm. (**I**–**P**) Fluorescence intensity profile analysis of TPXL proteins and microtubules. The blue lines indicate the regions selected for line-scan analysis. Relative fluorescence intensity profiles of mCitrine-TPXL proteins and tdTomato-TUA5 along the indicated axis were plotted to evaluate the spatial distribution of TPXL proteins on phragmoplast microtubules.

To further investigate the localization behavior of TPXL proteins during cell division, mitotic cells were induced by the co-expression of AtCYCD3;1 (Cyclin D3;1), which promotes cell cycle progression and increases the frequency of dividing cells [21]. During metaphase, most TPXL proteins displayed a uniform distribution along spindle microtubules. However, TPXL3 showed a distinct localization pattern, with accumulation toward the spindle poles (Figure 3). During cytokinesis, all eight TPXL proteins localized to the phragmoplast microtubule arrays (Figure 4). Interestingly, TPXL2 and TPXL3 concentrated at or near the distal ends of the phragmoplast microtubules, whereas the remaining TPXL members were distributed more uniformly along the phragmoplast microtubules. These observations propose that although TPXL proteins may associate with microtubules of the spindle and the phragmoplast in this system, individual family members exhibit differential spatial distributions during mitosis.

Together, these results suggest that *Arabidopsis* TPXL proteins support the broad microtubule association property of the TPX2 family during both interphase and cell division. However, several TPXL members exhibit distinct localization patterns during mitosis, implying potential functional differences among TPXL family members during plant cell division, although direct microtubule binding and the endogenous localization patterns of these proteins in *Arabidopsis* remain to be established.

### 2.3. Structural Prediction Reveals Divergent Aurora Kinase Association Among Arabidopsis TPXL Proteins

In animals, TPX2 plays a critical role in mitotic spindle assembly through its interaction with and activation of Aurora A. TPX2 and Aurora A kinase form a conserved regulatory module in which their reciprocal interaction and phosphorylation events coordinate spindle organization during mitosis [22,23]. To investigate whether *Arabidopsis* TPXL proteins retain the potential to associate with Aurora kinases, we performed AlphaFold3-based structural prediction between the TPXL1–TPXL8 proteins and the three *Arabidopsis* Aurora kinases (AUR1, AUR2, and AUR3). The predicted structural models proposed distinct interaction propensities among different TPXL members. Notably, TPXL2, TPXL3, TPXL4, and TPXL8 exhibited substantially higher predicted interface confidence scores (ipTM and pTM values) with Aurora kinases compared with TPXL1, TPXL5, TPXL6, and TPXL7 (Figure S3). These results suggest that Aurora kinase association is not uniformly conserved among TPXL family members but rather exhibits potential functional diversification within the TPXL subfamily. Based on the higher predicted confidence scores, TPXL2, TPXL3, TPXL4, and TPXL8 were selected for more detailed structural analysis of TPXL–Aurora association.

**Figure 5:**
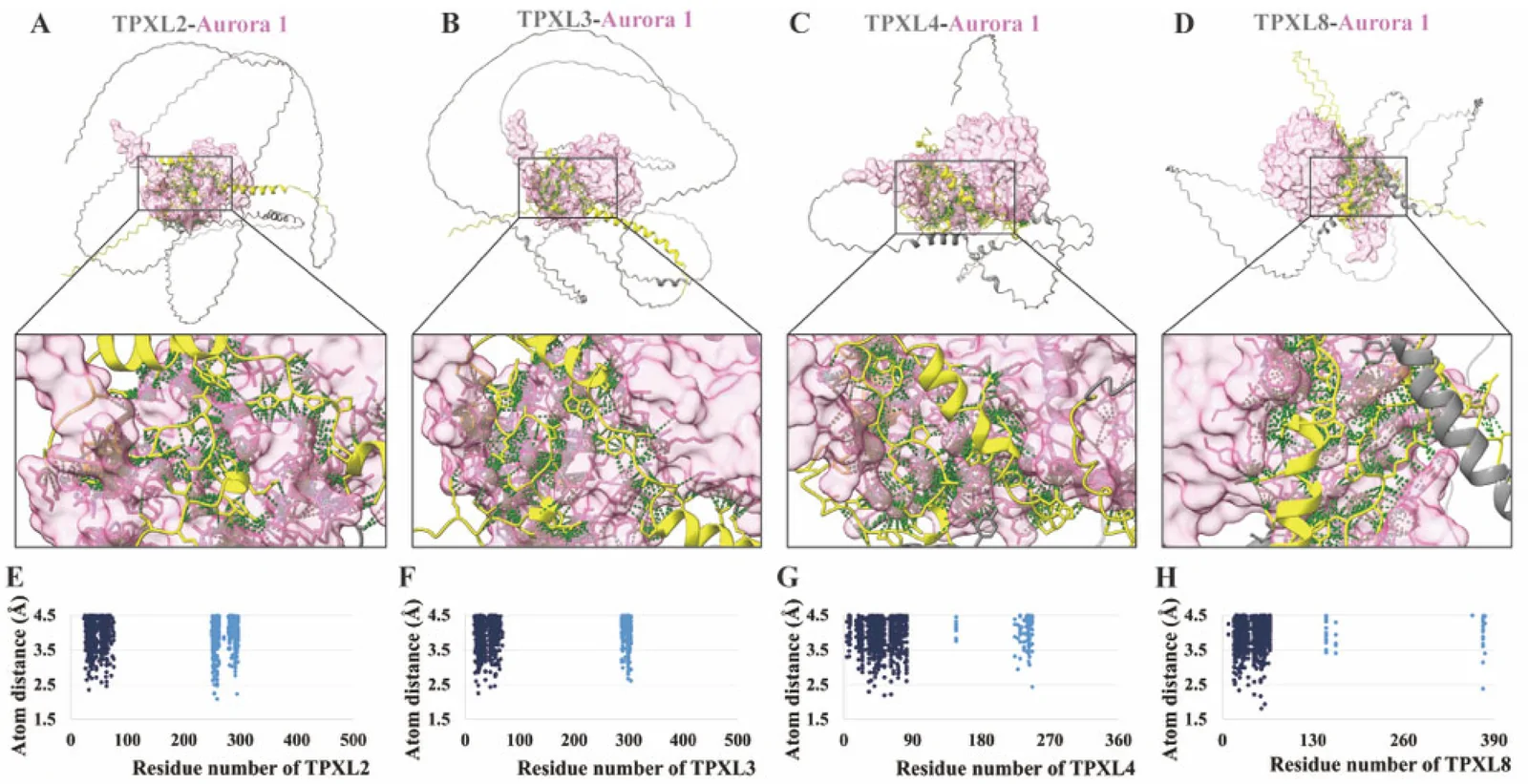
AlphaFold3 predicts Aurora-binding domain-mediated association between TPXL proteins and AUR1. (**A**–**D**) Predicted structural models of TPXL2, TPXL3, TPXL4, and TPXL8 proteins in complex with *Arabidopsis* AUR1 generated using AlphaFold3. TPXL proteins are shown in gray, with the conserved Aurora-binding domains highlighted in yellow. AUR1 is shown in pink. Predicted atomic contacts between TPXL proteins and Aurora kinases with interatomic distances shorter than 4.5 Å are indicated by green dashed lines. (**E**–**H**) Distribution of predicted atomic contacts between TPXL proteins and AUR1. The *x*-axis represents the amino acid positions of TPXL proteins, and the *y*-axis indicates the corresponding interatomic distances between TPXL and AUR1 atoms. Contacts within the Aurora-binding domains are shown as dark blue dots, whereas contacts outside the Aurora-binding domains are shown as light blue dots.

Previous studies have identified an N-terminal Aurora-binding domain in specific TPXL proteins [14]. Based on these annotations, the predicted Aurora-binding domains of TPXL2, TPXL3, TPXL4, and TPXL8 were highlighted in yellow in the structural models (Figure 5A–D and Figure S4A–D,I–L). To further characterize the structural basis of TPXL–Aurora association, atomic contacts between TPXL proteins and Aurora kinases were analyzed using an interatomic distance cutoff of 4.5 Å. The contacting atoms were mapped onto TPXL protein sequences, with Aurora-binding domain-mediated contacts highlighted in dark blue (Figure 5E–H and Figure S4E–H,M–P). For predicted contacts with AUR1, the Aurora-binding domains accounted for 60.7%, 77.0%, 90.1%, and 95.9% of the total predicted TPXL–AUR1 contacts in TPXL2, TPXL3, TPXL4, and TPXL8, respectively. Similarly, these domains contributed 69.4%, 62.4%, 78.7%, and 97.2% of the predicted contacts with AUR2, and 100%, 91.6%, 87.4%, and 76.4% of the contacts with AUR3 (Figure 5E–H and Figure S4E–H,M–P). These results suggest that the annotated Aurora-binding regions make substantial contributions to the predicted interfaces in these structural modules mediating the predicted association between TPXL proteins and Aurora kinases. Furthermore, analysis of the PAE maps showed that the predicted contacting regions between TPXL2, TPXL3, TPXL4, TPXL8, and Aurora kinases exhibited relatively low prediction errors, represented by high-confidence regions in dark green (Figure S5), further supporting the reliability of the predicted TPXL–Aurora interaction interfaces.

Together, these predicted structural analyses suggest that *Arabidopsis* TPXL proteins have divergent capacities for Aurora kinase association. TPXL2, TPXL3, TPXL4, and TPXL8, selected as the strongest candidates for experimental testing, provide a structural basis for potential Aurora kinase interaction, whereas other TPXL members exhibit weaker predicted associations, suggesting functional specialization within the TPXL family. These predictions were treated as structural hypotheses and evaluated further using experimental interaction assays.

### 2.4. TPXL2, TPXL3, TPXL4, and TPXL8 Detectably Interact with AUR1 and AUR2 but Not AUR3

**Figure 6:**
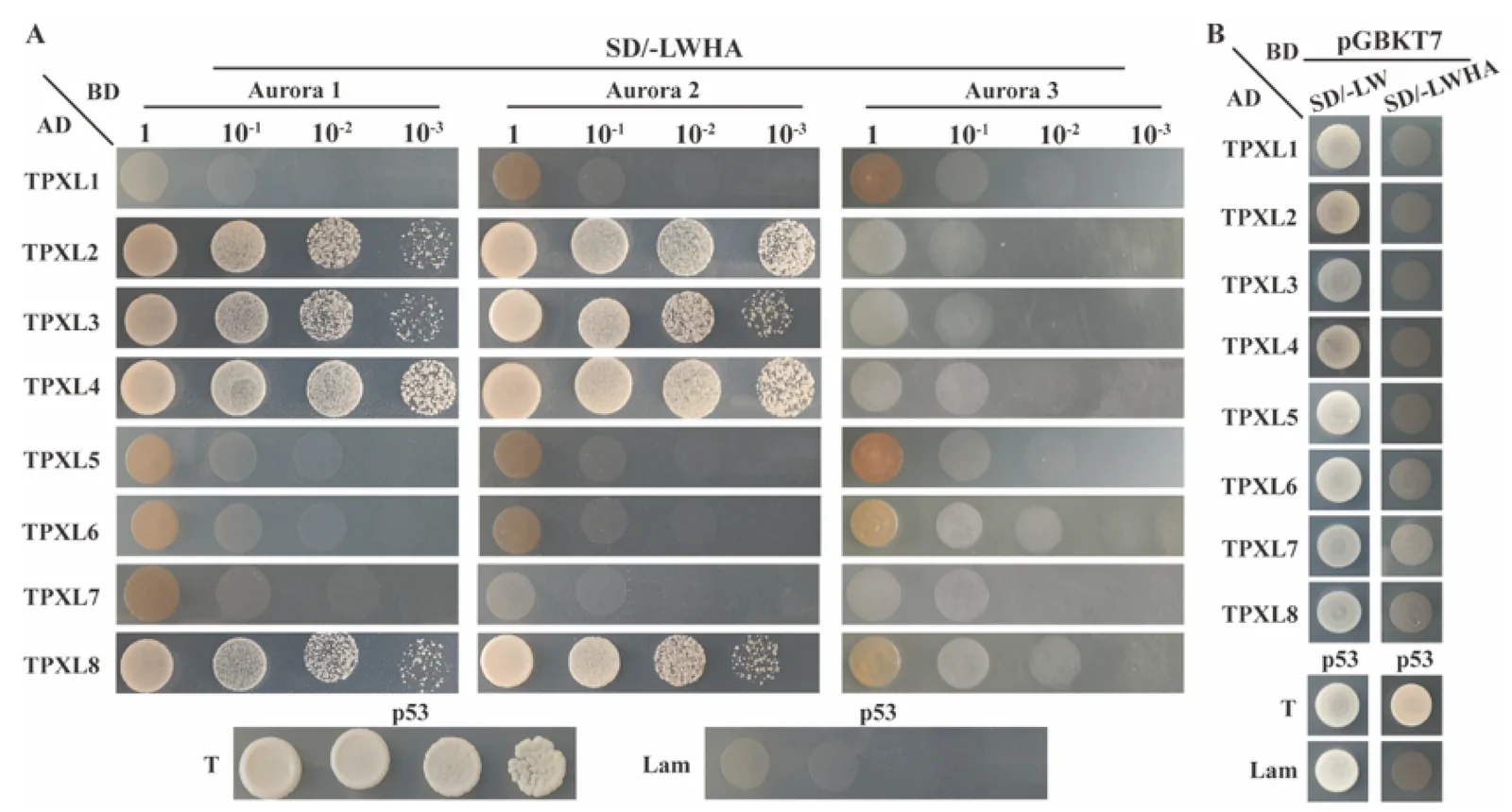
TPXL2, TPXL3, TPXL4, and TPXL8 interact with *Arabidopsis* Aurora kinases AUR1 and AUR2. (**A**) Y2H assays were performed to examine interactions between the TPXL1–TPXL8 proteins and *Arabidopsis* Aurora kinases AUR1, AUR2, and AUR3. TPXL proteins were fused with the GAL4 activation domain (AD), and Aurora kinases were fused with the GAL4 DNA-binding domain (BD). TPXL2, TPXL3, TPXL4, and TPXL8 showed interactions with AUR1 and AUR2, whereas no detectable interaction was observed between TPXL proteins and AUR3. (**B**) Autoactivation assays were performed to evaluate the transcriptional activation activity of the TPXL1–TPXL8 proteins in yeast. Yeast cells co-transformed with AD-TPXL1–8 and BD-empty vector were grown on SD/-Leu/-Trp (SD/-LW) medium and SD/-Leu/-Trp/-His/-Ade (SD/-LWHA) medium. None of the TPXL1–TPXL8 proteins exhibited self-activation activity. The T-p53 pair was used as a positive control, and the Lam-p53 pair was used as a negative control. All experiments were repeated three times.

Based on the AlphaFold3-predicted association between TPXL proteins and Aurora kinases, we next experimentally examined whether TPXL members interact with *Arabidopsis* Aurora kinase proteins. Yeast two-hybrid (Y2H) assays were performed to assess interactions between TPXL1–TPXL8 and the three *Arabidopsis* Aurora kinases, AUR1, AUR2, and AUR3. Consistent with the structural prediction, Y2H assays revealed that TPXL2, TPXL3, TPXL4, and TPXL8 interacted with both AUR1 and AUR2, whereas the remaining TPXL members showed no detectable interaction with these Aurora kinases (Figure 6). Interestingly, although AlphaFold3 predicted potential associations between TPXL proteins and AUR3, none of the eight TPXL members exhibited any detectable interaction with AUR3 in the Y2H assay (Figure 6), indicating that predicted structural compatibility does not necessarily reflect productive interaction *in vivo*.

**Figure 7:**
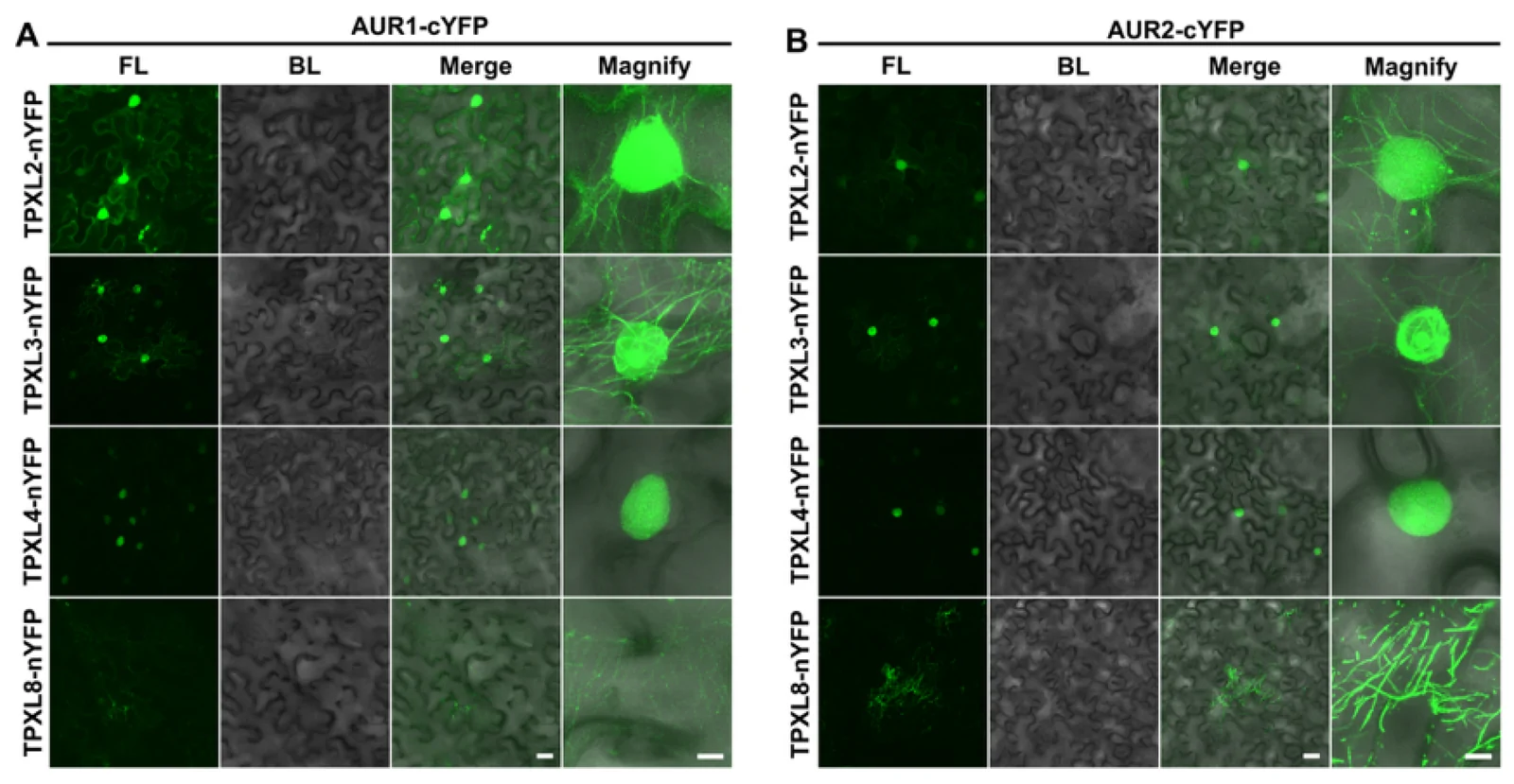
TPXL–Aurora complexes exhibit distinct subcellular localization patterns revealed by the BiFC assays. (**A**,**B**) The interactions were verified between TPXL2/TPXL3/TPXL4/TPXL8 and AUR1/2 by BiFC assays, respectively. BiFC assays were performed in *Nicotiana benthamiana* leaf epidermal cells to confirm TPXL–Aurora interactions. Reconstituted YFP fluorescence indicates interaction between TPXL proteins and Aurora kinases. TPXL2–AUR, TPXL3–AUR, TPXL4–AUR, and TPXL8–AUR complexes (green) displayed distinct intracellular localization patterns. FL: Fluorescence, BF: Brightfield. All experiments were repeated three times, and at least 3 cells were analyzed each time. Scale bars, 20 μm (FL, BL, and Merge) and 5 μm (Magnify).

To further validate the TPXL–Aurora associations and examine their subcellular localization patterns, bimolecular fluorescence complementation (BiFC) assays were performed in *Nicotiana benthamiana* leaf epidermal cells. The BiFC results confirmed the interactions between TPXL2, TPXL3, TPXL4, and TPXL8 and Aurora kinases (AUR1, AUR2, but not AUR3) (Figure 7 and Figure S6), further supporting the Y2H observations. Interestingly, the BiFC signals revealed distinct subcellular distributions among different TPXL–Aurora complexes, suggesting potential functional diversification. The TPXL2–AUR1/2 complex displayed diffuse fluorescence throughout the nucleus, accompanied by filamentous signals surrounding the nuclear envelope region. The TPXL3–AUR1/2 complex exhibited prominent filamentous localization within the nucleus, with enhanced accumulation in the nucleolus, and additional filamentous signals throughout the cytoplasm. In contrast, TPXL4–AUR1/2 interaction signals were exclusively detected in the nucleus and showed a diffuse distribution pattern. The TPXL8–AUR1/2 complex was predominantly detected in the cytoplasm, displaying filamentous localization patterns (Figure 7).

Together, these results support detectable interactions of TPXL2, TPXL3, TPXL4, and TPXL8 with AUR1 and AUR2 under the conditions tested. The absence of detectable TPXL–AUR3 interaction also illustrates that structural compatibility predicted by AlphaFold3 does not necessarily correspond to an experimentally detectable protein association.

### 2.5. AUR1 Localizes to Spindle and Phragmoplast Microtubules with TPXL-Associated Patterns During Cell Division

Given that TPXL2, TPXL3, TPXL4, and TPXL8 interact with both Aurora kinases and are associated with tubulin heterodimer, we next investigated whether TPXL proteins could serve as a potential structural linker between Aurora kinase and microtubules. AlphaFold3 was used to predict ternary complex models consisting of TPXL2/3/4/8, AUR1, and the tubulin heterodimer TUA5/TUB6.

The predicted ternary complexes showed moderate confidence scores, with ipTM and pTM values ranging from 0.5 to 0.65 (Figure S7A). These models therefore provide a potential structural framework for TPXL–AUR1–tubulin association, although they are interpreted as predicted molecular arrangements rather than experimentally validated complexes. In these models, TPXL proteins occupied a central position between Aurora kinase and tubulin. Specifically, the conserved TPX2 domains of TPXL2, TPXL3, TPXL4, and TPXL8 maintained predicted associations with the tubulin heterodimer, whereas their N-terminal regions formed contacts with AUR1 (Figure S7B–E), consistent with the previously identified tubulin-associated and Aurora-binding regions. To further evaluate whether AUR1 possesses an intrinsic capacity to associate with tubulin independently of TPXL, we generated a separate AlphaFold3 prediction model containing AUR1 and the TUA5/TUB6 heterodimer without TPXL proteins. In this binary model, no predicted atomic contacts were observed between AUR1 and TUA5, whereas only a very limited number of atomic contacts were detected between AUR1 and TUB6 within the 4.5 Å cutoff (Figure S8). These results suggest that AUR1 alone lacks a prominent predicted tubulin-binding interface, and that its association with microtubule structures may be facilitated by TPXL proteins.

**Figure 8:**
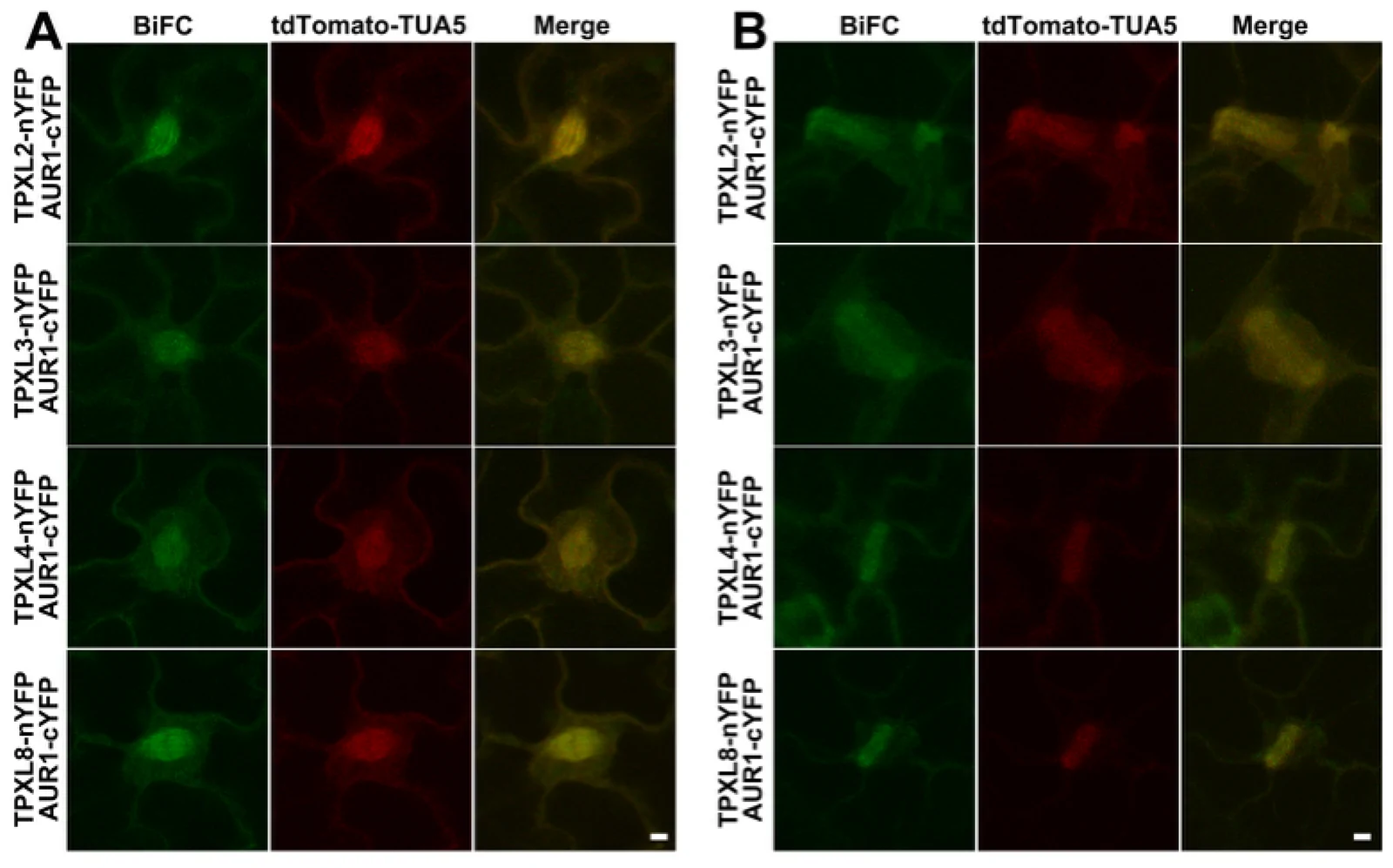
TPXL–Aurora complexes exhibit subcellular localization during cell division revealed by BiFC assays. (**A**,**B**) The interactions were verified between TPXL2/TPXL3/TPXL4/TPXL8 and AUR1 during cell division by BiFC assays, respectively. BiFC assays were performed in *Nicotiana benthamiana* leaf epidermal cells to confirm TPXL–Aurora interactions. Reconstituted YFP fluorescence indicates interaction between TPXL proteins and Aurora kinases. TPXL2–AUR1, TPXL3–AUR1, TPXL4–AUR1, and TPXL8–AUR1 complexes (green) were transiently co-expressed with tdTomato-TUA5 (red) in *Nicotiana benthamiana* leaf epidermal cells. Mitotic cells were induced by the co-expression of AtCYCD3;1. During metaphase, TPXL–AUR1 complexes localized along the spindle microtubules. During cytokinesis, TPXL–AUR1 complexes accumulated at the phragmoplast microtubule array. All experiments were repeated three times, and at least 3 cells were analyzed each time. Scale bars, 5 μm.

To explore whether interactions of TPXL proteins and Aurora kinase are on microtubule arrays during cell division, we observed the subcellular localization of TPXL–AUR1 complexes and TUA5-labled microtubule arrays during cell division in *Nicotiana benthamiana* leaf epidermal cells, and dividing cells were induced by the co-expression of AtCYCD3;1. During metaphase, TPXL–AUR1 complexes localized along the spindle microtubules. During cytokinesis, TPXL–AUR1 complexes accumulated at the phragmoplast microtubule arrays (Figure 8). These data suggest that TPXL proteins interact with AUR1 on specific microtubule arrays during cell division in this transient-expression system.

To further investigate whether TPXL proteins and Aurora kinase have similar spatial distributions during cell division, we examined the subcellular localization pattern of AUR1 in the presence of TPXL2, TPXL3, TPXL4, or TPXL8. AUR1-mTurquoise2 was transiently co-expressed with mCitrine-TPXL2, TPXL3, TPXL4, or TPXL8 in *Nicotiana benthamiana* leaf epidermal cells, and dividing cells were induced by the co-expression of AtCYCD3;1. During metaphase, AUR1 displayed a microtubule-associated distribution pattern along the entire spindle apparatus, overlapping with the localization patterns observed for TPXL proteins (Figure S9). During cytokinesis, AUR1 accumulated at the phragmoplast microtubule array, similar to TPXL2, TPXL3, TPXL4, and TPXL8 (Figure S9). The comparable localization patterns of AUR1 and TPXL proteins during different stages of cell division are consistent with the predicted TPXL-mediated association between Aurora kinase and microtubules. Together with the structural prediction and protein interaction analyses, these results suggest that TPXL proteins may associate with AUR1 and microtubule arrays during plant cell division.

## 3. Discussion

### 3.1. Conserved Microtubule Association and Distinct Localization Patterns Among Arabidopsis TPXL Proteins

TPX2 is a conserved microtubule-associated protein that plays essential roles in spindle assembly and chromosome segregation by regulating microtubule organization and Aurora kinase activity [10]. In animals, TPX2 serves as a key spindle assembly factor by targeting Aurora A to spindle microtubules and promoting its activation [13]. Although TPX2-like proteins have been identified in plants, the extent to which plant TPXL proteins retain conserved TPX2 functions and whether individual members have acquired specialized roles remain largely unclear [24].

In this study, we suggest that the eight *Arabidopsis* TPXL proteins share a conserved capacity for microtubule association but exhibit functional diversification during cell division. AlphaFold3-based structural prediction revealed that the conserved TPX2 domain presented in all TPXL proteins contributes to predicted tubulin association. Consistently, transient expression analyses in *Nicotiana benthamiana* leaf epidermal cells showed that TPXL1–TPXL8 localized to cortical microtubules during interphase, indicating that microtubule association represents a conserved feature of the TPXL family. Despite this conserved property, TPXL proteins displayed distinct localization patterns during mitosis. Most TPXL members exhibited uniform localization along spindle microtubules, whereas TPXL3 accumulated at spindle poles. During cytokinesis, all TPXL proteins localized to phragmoplast microtubules, but TPXL2 and TPXL3 showed enrichment at or near the distal ends of the phragmoplast microtubules. These differences suggest that TPXL members may have distinct functions in microtubule arrays during cell division, but the current data do not establish the molecular basis of those differences, and endogenous localization and regulation in *Arabidopsis* need be verified in future studies.

The coexistence of conserved microtubule association and divergent mitotic localization patterns suggests that the TPXL family has undergone functional diversification during evolution [25,26]. While the conserved TPX2 domain may contribute to microtubule association throughout the TPXL family, additional regulatory regions may determine the distinct cellular roles of TPXL proteins. This speculation needs to be verified through experiments in the future.

### 3.2. Aurora-Binding Activity Underlies Functional Diversification Within the Arabidopsis TPXL Family

A major finding of this study is that Aurora kinase association is not uniformly conserved among *Arabidopsis* TPXL proteins. In animals, the TPX2–Aurora A module represents a fundamental regulatory mechanism that couples Aurora kinase activation with spindle microtubule organization [12,27,28]. However, our structural and experimental analyses proposed that only a subset of *Arabidopsis* TPXL proteins, including TPXL2, TPXL3, TPXL4, and TPXL8, retain detectable Aurora-binding activity. AlphaFold3 predictions suggested that TPXL2, TPXL3, TPXL4, and TPXL8 possess higher structural compatibility with Aurora kinases compared with other TPXL members. Detailed structural analyses further revealed that the N-terminal Aurora-binding domains of these proteins contribute substantially to the predicted Aurora-contacting interfaces. Consistent with these predictions, Y2H and BiFC assays confirmed that TPXL2, TPXL3, TPXL4, and TPXL8 interacted with AUR1 and AUR2.

In contrast, TPXL1, TPXL5, TPXL6, and TPXL7 displayed no detectable interaction with Aurora kinases despite retaining the conserved TPX2 domain and microtubule localization capability. These findings suggest that microtubule association and Aurora-binding capacity are different in the plant TPXL family. The conserved TPX2 domain may provide a shared structural basis for microtubule association, whereas Aurora-binding activity has been selectively maintained in specific TPXL members. Such diversification may enable individual TPXL proteins to perform distinct functions during cell division. TPXL proteins retaining Aurora-binding activity may coordinate microtubule organization with Aurora kinase, whereas other members may function primarily as microtubule-associated factors independent of Aurora kinase.

### 3.3. Structural Prediction and Experimental Validation Uncover the Importance of Cellular Context for TPXL–Aurora Interaction

An interesting observation of this study is the discrepancy between AlphaFold3 prediction and experimental validation regarding TPXL–Aurora interactions. Although AlphaFold3 predicted potential associations between several TPXL proteins and AUR3, neither the Y2H nor BiFC assays detected interactions between any TPXL members and AUR3. This discrepancy highlights that structural compatibility predicted by artificial intelligence does not necessarily correspond to biologically relevant protein associations. AlphaFold3 predicts possible biomolecular complex conformations based on learned structural and interaction patterns; however, the occurrence of protein interactions *in vivo* is additionally influenced by multiple biological constraints, including subcellular localization, temporal expression patterns, conformational flexibility, post-translational modifications, and the availability of regulatory cofactors. Therefore, the predicted TPXL–AUR3 complexes may represent structurally feasible configurations that are not adopted under physiological conditions.

The distinct cellular functions and localization patterns of *Arabidopsis* Aurora kinases may provide another explanation for the differential TPXL-binding specificity. In *Arabidopsis*, AUR1 and AUR2 are primarily associated with mitotic microtubule structures, including spindle and phragmoplast, where they contribute to the regulation of cell division [29,30,31]. In contrast, AUR3 exhibits distinct localization characteristics and has been associated with centromeric regions and chromosome-related processes including regulation of the centromeric histone variant CENH3 [9]. The spatial separation of AUR3 from microtubule-based mitotic structures suggests that AUR3 may function within a regulatory pathway distinct from the TPXL-mediated microtubule-associated module.

Consistent with this speculation, our results suggest that TPXL proteins associate with microtubule structures through their conserved TPX2 domains and interact with AUR1 through specific Aurora-binding regions. Thus, the absence of TPXL–AUR3 interaction may not reflect an inability of TPXL proteins to recognize Aurora kinases, but rather differences in cellular targeting requirements and regulatory contexts. AUR3 may instead rely on alternative centromere-associated factors for its localization and function during chromosome segregation.

Together, these findings imply that Aurora kinase family members have undergone potential functional diversification in plants. While AUR1 and AUR2 appear to be incorporated into a TPXL-dependent microtubule association, AUR3 represents a specialized Aurora kinase associated with centromere-related functions. The divergence between predicted and experimentally validated interactions further emphasizes that protein interaction networks are determined not only by structural compatibility, but also by cellular context. Compared with previous studies focusing primarily on TPXL3 or the general functional divergence of TPX2 family proteins, our study provides a family-wide view of TPXL functional diversification and reveals that microtubule association is broadly conserved, whereas Aurora kinase association has been selectively retained in specific members.

### 3.4. TPXL Proteins May Connect Aurora Kinase with Microtubule Arrays

The interaction between TPX2 and Aurora kinase is a central mechanism regulating spindle assembly in animals. TPX2 recruits Aurora A to spindle microtubules, promotes Aurora A activation, and undergoes reciprocal regulation through phosphorylation [13]. Our structural analyses provide the potential relationship that *Arabidopsis* TPXL proteins may couple Aurora kinase with microtubule organization. Predicted TPXL–AUR1–tubulin ternary complexes suggested that TPXL proteins occupy a central position between Aurora kinase and tubulin. The TPX2 domain mediates predicted association with tubulin, and the N-terminal Aurora-binding domain is predicted to contact AUR1. Importantly, predictive direct structural contacts between AUR1 and tubulin were limited, suggesting that Aurora kinase may associate with microtubule structures primarily through TPXL mediation. Consistent with this model, TPXL proteins interact with AUR1 on microtubule arrays of spindle and phragmoplast during cell division. In addition, AUR1 displayed localization patterns similar to TPXL proteins during cell division. AUR1 localized along spindle microtubules during metaphase and accumulated at the phragmoplast during cytokinesis, matching the microtubule-associated distribution of TPXL2, TPXL3, TPXL4, and TPXL8.

Together, these findings suggest that TPXL proteins may link Aurora kinase and microtubule structures. Through TPXL proteins interacting with Aurora kinase and associated with tubulin, TPXL proteins and Aurora may synergistically play a role within specific microtubule arrays during plant cell division.

### 3.5. Selective Aurora Interaction with TPXL Family Members

Although the TPX2–Aurora regulatory module is conserved across eukaryotes, plants possess distinct cellular architectures and rely on acentrosomal microtubule arrays for spindle and phragmoplast. The diversification of TPXL proteins may therefore represent an adaptation to plant-specific mechanisms of microtubule organization.

Our results suggest that *Arabidopsis* TPXL proteins preserve key characteristics of the TPX2–Aurora interaction while undergoing substantial functional diversification. All eight TPXL proteins retain the ability to associate with microtubules, whereas only a subset of TPXL members (TPXL2, TPXL3, TPXL4, and TPXL8) maintains Aurora-interacting capacity. These Aurora-interacting TPXL proteins may function as molecular adaptors that connect Aurora kinase with microtubule structures, similar to the role of TPX2 in animal cells [32].

However, unlike the relatively conserved TPX2–Aurora A relationship described in animals, the *Arabidopsis* TPXL family possibly exhibits distinct functional specialization. The differential Aurora-binding ability among TPXL members suggests that TPXL–Aurora interaction has been selective within the TPXL family. Moreover, the lack of detectable interaction between TPXL proteins and AUR3 further suggest that TPXL–Aurora interaction is selective.

Overall, our study suggests that *Arabidopsis* TPXL proteins preserve a conserved TPX2-like microtubule association while diversifying in their capacity to interact with Aurora kinases. These findings may establish TPXL proteins as important candidate factors connecting Aurora kinase pathways with microtubule organization and provide a new clue that different TPXL–Aurora complexes may perform distinct functions during plant cell division.

## 4. Materials and Methods

### 4.1. AlphaFold3-Based Protein Structure Prediction and Structural Analysis

Protein structure predictions were performed using the AlphaFold3 web server (accessed on 4 June 2026) [17]. The amino acid sequences of *Arabidopsis* TPXL1–TPXL8, tubulin subunits (TUA5, TUB6, TUA2, and TUB4), and Aurora kinases (AUR1, AUR2, and AUR3) were obtained from The *Arabidopsis* Information Resource (TAIR, https://www.arabidopsis.org (accessed on 21 April 2026)). The corresponding TAIR accession numbers and protein sequence information used for prediction are provided in Table S1. Protein sequences corresponding to the annotated reference isoforms were used for all AlphaFold3 predictions.

Protein complex models were generated by submitting the corresponding protein combinations to the AlphaFold3 web server. For each protein combination, five ranked structural models were generated by AlphaFold3. Candidate models were evaluated based on AlphaFold3 confidence metrics, including ipTM, pTM, PAE, and pLDDT values. The ipTM score was used as an indicator of confidence in predicted inter-chain interfaces, whereas the pTM score was interpreted as an estimate of the overall confidence of the predicted complex structure rather than as a direct measure of protein–protein interaction strength. PAE plots were further examined to assess the confidence of relative domain orientations and inter-chain arrangements. To investigate potential TPXL–tubulin associations, each TPXL protein was modeled with two different α/β-tubulin heterodimers, TUA5/TUB6 and TUA2/TUB4. Because both tubulin combinations produced comparable prediction confidence, TUA5/TUB6 was selected for subsequent structural analysis. For Aurora kinase interaction analysis, TPXL1–TPXL8 were individually modeled with AUR1, AUR2, and AUR3. Based on the prediction confidence and interaction scores, TPXL2, TPXL3, TPXL4, and TPXL8 were further analyzed in complex with AUR1 and TUA5/TUB6. The amino acid boundaries of the TPX2 domain and Aurora-binding domain were obtained from the domain annotations provided by TAIR and are summarized in Table S2. These annotated regions were highlighted in the AlphaFold3-predicted structural models and used for subsequent atomic contact analyses.

The AlphaFold3-generated structural models were subsequently analyzed and visualized using UCSF ChimeraX version 1.11.1. Protein structures were displayed using different colors to distinguish individual proteins and annotated functional domains including the TPX2 domain and Aurora-binding domain (Table S2). Potential intermolecular contacts were identified by measuring atom–atom distances between interacting proteins, and atomic pairs within 4.5 Å were considered potential interaction sites. The spatial distribution of these atomic contacts was visualized in structural models and plotted according to the TPXL amino acid positions. Contacts associated with annotated functional domains were distinguished from those outside the domains. The contribution of each domain to the predicted interaction interface was calculated as the proportion of domain-associated atomic contacts relative to the total number of intermolecular contacts.

### 4.2. Plant Materials and Agrobacterium-Mediated Transient Expression

*Nicotiana benthamiana* plants were grown under long-day conditions (16 h light/8 h dark) at 22–25 °C. Four- to six-week-old plants were used for Agrobacterium-mediated transient transformation.

The coding sequences of TPXL1–TPXL8 were cloned into the binary vector *pEG101-pUBQ14::mCitrine-TPXL* for protein localization analysis. Aurora kinase constructs were generated using *pCAMBIA1300-pRPS5A::AUR-mTurquoise2*. The microtubule marker construct *PGWB2-pUBQ14::tdTomato-TUA5* was used to visualize the microtubule arrays. The primers are shown in Table S3.

*Agrobacterium tumefaciens* strain GV3101 (AC1001; Shanghai Weidi Biotechnology Co., Ltd., Shanghai, China) carrying the indicated constructs was infiltrated into *Nicotiana benthamiana* leaves. Fluorescence signals were analyzed 48–72 h after infiltration.

To induce mitotic progression in tobacco epidermal cells, AtCYCD3;1 was transiently co-expressed as previously described [21,33].

### 4.3. Confocal Microscopy Analysis

Fluorescence imaging was performed using confocal laser scanning microscopy Olympus FV3000RS (Olympus Corporation, Tokyo, Japan) equipped with a 60× objective lens. YFP/mCitrine, tdTomato, and mTurquoise2 fluorescence signals were detected using excitation wavelengths of 514 nm, 561 nm, and 445 nm, respectively.

For interphase localization analysis, mCitrine-tagged TPXL proteins were observed together with tdTomato-TUA5-labeled cortical microtubules. For mitotic localization analysis, cells expressing AtCYCD3;1 were examined to determine TPXL localization patterns during spindle assembly and cytokinesis.

For Aurora localization analysis, AUR-mTurquoise2 was co-expressed with TPXL proteins, and fluorescence distribution during mitosis was analyzed by confocal laser scanning microscopy Olympus FV3000RS (Olympus Corporation, Tokyo, Japan).

### 4.4. Yeast Two-Hybrid Assay

Yeast two-hybrid assays were performed using the GAL4-based system. Full-length coding sequences of TPXL proteins were cloned into the prey vector *pGADT7*, whereas Aurora kinase coding sequences were cloned into the bait vector *pGBKT7*. The primers are shown in Table S3.

The resulting plasmids were co-transformed into *Saccharomyces cerevisiae* strain AH109. Transformants were selected on synthetic defined medium lacking leucine and tryptophan (SD/-Leu/-Trp). Protein interactions were evaluated by growth on selective medium lacking leucine, tryptophan, histidine, and adenine (SD/-Leu/-Trp/-His/-Ade).

The interaction between T-antigen and p53 (T-p53) was used as a positive control, and Lam-p53 was used as a negative control.

### 4.5. Bimolecular Fluorescence Complementation (BiFC) Assay

BiFC assays were performed using the split-YFP system. TPXL and Aurora coding sequences were fused with complementary fragments of YFP (nYFP or cYFP), respectively. The primers are shown in Table S3. The resulting constructs were introduced into *Agrobacterium tumefaciens* GV3101 and transiently expressed in *Nicotiana benthamiana* epidermal cells.

YFP fluorescence resulting from TPXL–Aurora interactions was detected by confocal laser scanning microscopy. The subcellular localization patterns of reconstituted fluorescence signals were analyzed during interphase and cell division.

## 5. Conclusions

In this study, we investigated the structural and functional characteristics of the *Arabidopsis* TPXL family and revealed both conserved and diversified features among TPXL members. AlphaFold3-based structural analyses combined with subcellular localization studies suggested that all eight TPXL proteins retained microtubule-associated properties, with the conserved TPX2 domain representing a potential important structure supporting their association with tubulin and microtubule arrays.

In contrast, Aurora kinase association has undergone functional diversification among TPXL members. Only TPXL2, TPXL3, TPXL4, and TPXL8 retained detectable interactions with AUR1 and AUR2, whereas other TPXL proteins lacked Aurora-interacting activity despite maintaining microtubule localization. Structural modeling, together with biochemical and cellular validation, further suggests that Aurora-interacting TPXL proteins may serve as molecular adaptors that connect Aurora kinase signaling with specific microtubule structures. The absence of TPXL–AUR3 interaction highlights the importance of cellular context. The current results are a structural and cellular framework for future mechanistic testing.

Collectively, our study expands the understanding of plant TPX2-like proteins by suggesting that the *Arabidopsis* TPXL family has retained a conserved TPX2-like microtubule association while undergoing diversification in TPXL–Aurora interaction. These findings provide a new clue that TPXL–Aurora complexes may perform different functions during plant cell division.

## Data Availability

The original contributions presented in this study are included in the article/Supplementary Materials. Further inquiries can be directed to the corresponding authors.

## Supplementary Materials

The following supporting information can be downloaded at: https://www.mdpi.com/article/10.3390/ijms27187980/s1.

## References

[1] Lipka E., Herrmann A., Mueller S. (2015). Mechanisms of plant cell division. Wiley Interdiscip. Rev. Dev. Biol..

[2] Rasmussen C.G., Wright A.J., Müller S. (2013). The role of the cytoskeleton and associated proteins in determination of the plant cell division plane. Plant J..

[3] Livanos P., Müller S. (2019). Division plane establishment and cytokinesis. Annu. Rev. Plant Biol..

[4] Wasteneys G.O. (2004). Progress in understanding the role of microtubules in plant cells. Curr. Opin. Plant Biol..

[5] Vavrdová T., Samaj J., Komis G. (2019). Phosphorylation of plant microtubule-associated proteins during cell division. Front. Plant Sci..

[6] Pais E., Schou K.B. (2026). Microtubule-Associated Proteins: From Dynamic Regulation of Microtubules to Cellular Architecture. Cells.

[7] Carmena M., Earnshaw W.C. (2003). The cellular geography of aurora kinases. Nat. Rev. Mol. Cell Biol..

[8] Vader G., Lens S.M. (2008). The Aurora kinase family in cell division and cancer. Biochim. Biophys. Acta (BBA) -Rev. Cancer.

[9] Demidov D., Heckmann S., Weiss O., Rutten T., Dvořák Tomaštíková E., Kuhlmann M., Scholl P., Municio C., Lermontova I., Houben A. (2019). Deregulated phosphorylation of CENH3 at Ser65 affects the development of floral meristems in *Arabidopsis thaliana*. Front. Plant Sci..

[10] Wittmann T., Wilm M., Karsenti E., Vernos I. (2000). TPX2, A novel *Xenopus* MAP involved in spindle pole organization. J. Cell Biol..

[11] Gruss O.J., Wittmann M., Yokoyama H., Pepperkok R., Kufer T., Silljé H., Karsenti E., Mattaj I.W., Vernos I. (2002). Chromosome-induced microtubule assembly mediated by TPX2 is required for spindle formation in HeLa cells. Nat. Cell Biol..

[12] Kufer T.A., Silljé H.H., Körner R., Gruss O.J., Meraldi P., Nigg E.A. (2002). Human TPX2 is required for targeting Aurora-A kinase to the spindle. J. Cell Biol..

[13] Bayliss R., Sardon T., Vernos I., Conti E. (2003). Structural basis of Aurora-A activation by TPX2 at the mitotic spindle. Mol. Cell.

[14] Smertenko A., Clare S.J., Effertz K., Parish A., Ross A., Schmidt S. (2021). A guide to plant TPX2-like and WAVE-DAMPENED2-like proteins. J. Exp. Bot..

[15] Boruc J., Deng X., Mylle E., Besbrugge N., Van Durme M., Demidov D., Toma¡ tíková E.D., Tan T.-R.C., Vandorpe M., Eeckhout D. (2019). TPX2-LIKE PROTEIN3 is the primary activator of α-aurora kinases and is essential for embryogenesis. Plant Physiol..

[16] Deng X., Higaki T., Lin H.-H., Lee Y.-R.J., Liu B. (2025). The unconventional TPX2 family protein TPXL3 regulates α Aurora kinase function in spindle morphogenesis in *Arabidopsis*. Plant Cell.

[17] Abramson J., Adler J., Dunger J., Evans R., Green T., Pritzel A., Ronneberger O., Willmore L., Ballard A.J., Bambrick J. (2024). Accurate structure prediction of biomolecular interactions with AlphaFold 3. Nature.

[18] Liu Z., Schneider R., Kesten C., Zhang Y., Somssich M., Zhang Y., Fernie A.R., Persson S. (2016). Cellulose-microtubule uncoupling proteins prevent lateral displacement of microtubules during cellulose synthesis in *Arabidopsis*. Dev. Cell.

[19] Ferreira M.J., Silva J., Pinto S.C., Coimbra S. (2023). I Choose You: Selecting Accurate Reference Genes for qPCR Expression Analysis in Reproductive Tissues in *Arabidopsis thaliana*. Biomolecules.

[20] Liang Z., Huang J., Wang Y., Hua S., Jiang K. (2025). Diverse microtubule-binding repeats regulate TPX2 activities at distinct locations within the spindle. J. Cell Biol..

[21] Xu J., Lee Y.R.J., Liu B. (2020). Establishment of a mitotic model system by transient expression of the D-type cyclin in differentiated leaf cells of tobacco (*Nicotiana benthamiana*). New Phytol..

[22] Garrido G., Vernos I. (2016). Non-centrosomal TPX2-dependent regulation of the Aurora A kinase: Functional implications for healthy and pathological cell division. Front. Oncol..

[23] Polverino F., Mastrangelo A., Guarguaglini G. (2024). Contribution of AurkA/TPX2 overexpression to chromosomal imbalances and Cancer. Cells.

[24] Dvořák Tomaštíková E., Rutten T., Dvořák P., Tugai A., Ptošková K., Petrovská B., Van Damme D., Houben A., Doležel J., Demidov D. (2020). Functional divergence of microtubule-associated TPX2 family members in *Arabidopsis thaliana*. Int. J. Mol. Sci..

[25] Smertenko T., Turner G., Fahy D., Brew-Appiah R.A., Alfaro-Aco R., de Almeida Engler J., Sanguinet K.A., Smertenko A. (2020). *Brachypodium distachyon* MAP20 functions in metaxylem pit development and contributes to drought recovery. New Phytol..

[26] Qian Y., Wang X., Liu Y., Wang X., Mao T. (2023). HY5 inhibits lateral root initiation in *Arabidopsis* through negative regulation of the microtubule-stabilizing protein TPXL5. Plant Cell.

[27] Sobajima T., Kowalczyk K.M., Skylakakis S., Hayward D., Fulcher L.J., Neary C., Batley C., Kurlekar S., Roberts E., Gruneberg U. (2023). PP6 regulation of Aurora A–TPX2 limits NDC80 phosphorylation and mitotic spindle size. J. Cell Biol..

[28] Gruss O.J., Vernos I. (2004). The mechanism of spindle assembly: Functions of Ran and its target TPX2. J. Cell Biol..

[29] Demidov D., Van Damme D., Geelen D., Blattner F.R., Houben A. (2005). Identification and dynamics of two classes of aurora-like kinases in *Arabidopsis* and other plants. Plant Cell.

[30] Van Damme D., De Rybel B., Gudesblat G., Demidov D., Grunewald W., De Smet I., Houben A., Beeckman T., Russinova E. (2011). *Arabidopsis* α Aurora kinases function in formative cell division plane orientation. Plant Cell.

[31] Deng X., Xiao Y., Tang X., Liu B., Lin H. (2024). *Arabidopsis* α-Aurora kinase plays a role in cytokinesis through regulating MAP65-3 association with microtubules at phragmoplast midzone. Nat. Commun..

[32] Özlü N., Srayko M., Kinoshita K., Habermann B., O’Toole E.T., Müller-Reichert T., Schmalz N., Desai A., Hyman A.A. (2005). An essential function of the *C. elegans* ortholog of TPX2 is to localize activated aurora A kinase to mitotic spindles. Dev. Cell.

[33] Dewitte W., Scofield S., Alcasabas A.A., Maughan S.C., Menges M., Braun N., Collins C., Nieuwland J., Prinsen E., Sundaresan V. (2007). *Arabidopsis* CYCD3 D-type cyclins link cell proliferation and endocycles and are rate-limiting for cytokinin responses. Proc. Natl. Acad. Sci. USA.

