# Supplementary material for: Arabidopsis TPXL Proteins Mediate a Selective Aurora Kinase–Microtubule Association During Cell Division
Source: Int J Mol Sci. 2026 Sep 8;27(18):7980. doi: 10.3390/ijms27187980 (PMC13606213; doi:10.3390/ijms27187980)
Supplement: Supplementary file 1 [file ijms-27-07980-s001.zip › Supplementary Figures-TPXL-Revised manuscript-dpz2.pdf]

A

		TPXL1	TPXL2	TPXL3	TPXL4	TPXL5	TPXL6	TPXL7	TPXL8
TUA5+TUB6	iPTM	0.59	0.7	0.71	0.72	0.59	0.73	0.64	0.69
	PTM	0.66	0.66	0.66	0.72	0.67	0.81	0.72	0.69
TUA2+TUB4	iPTM	0.58	0.65	0.71	0.7	0.57	0.76	0.6	0.63
	PTM	0.66	0.65	0.67	0.71	0.66	0.83	0.71	0.69

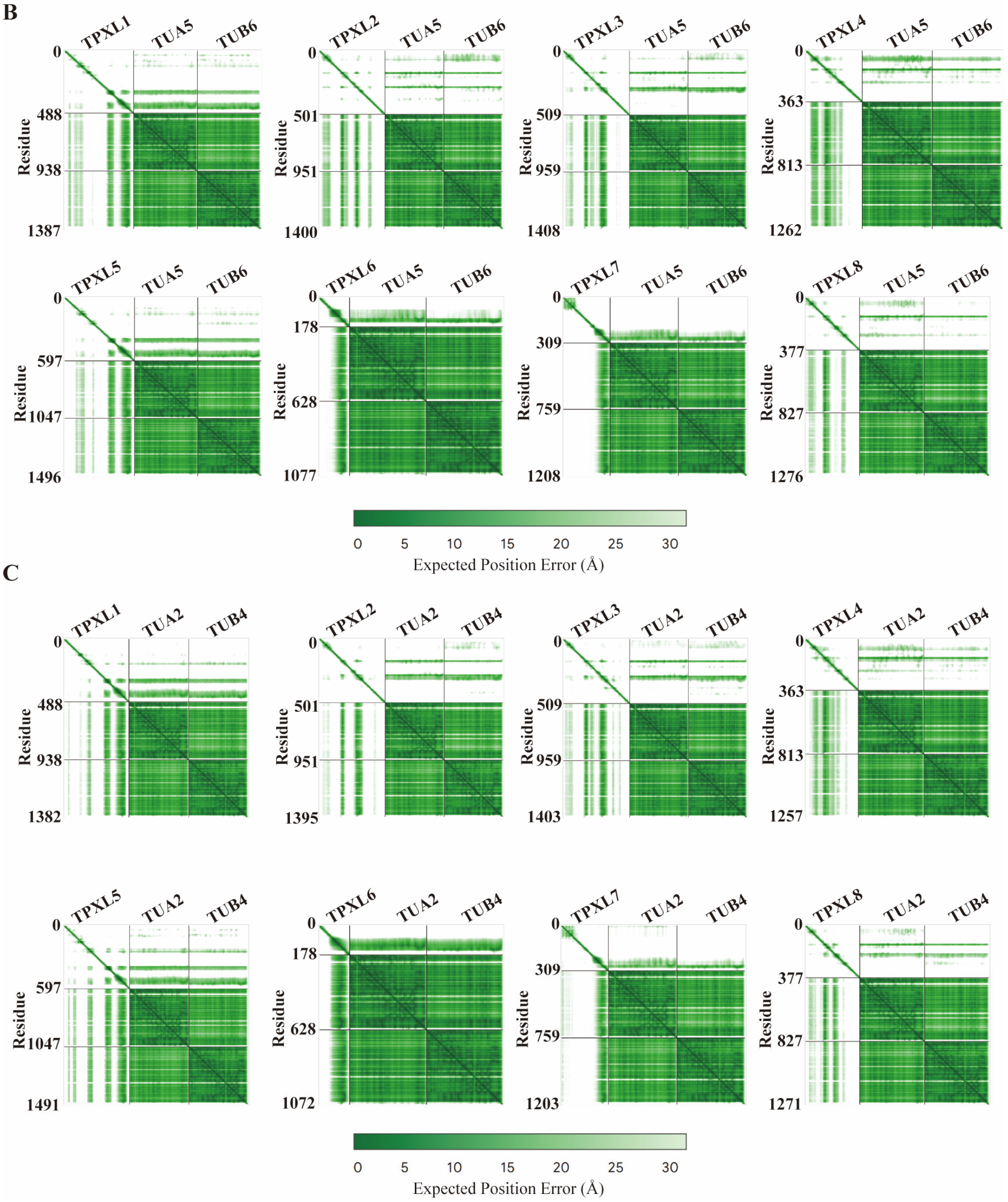

Figure S1. Confidence assessment of AlphaFold3-predicted TPXL–tubulin complex models. **(A)** Comparison of predicted interaction confidence scores for TPXL1–TPXL8 proteins in complex with two representative *Arabidopsis* tubulin heterodimers, TUA5/TUB6 and TUA2/TUB4. The ipTM scores and pTM scores are shown for each TPXL–tubulin complex. **(B)** PAE plots of TPXL1–TPXL8 proteins interacting with the TUA5/TUB6 tubulin heterodimer generated by AlphaFold3. **(C)** PAE plots of TPXL1–TPXL8 proteins interacting with the TUA2/TUB4 tubulin heterodimer generated by AlphaFold3.

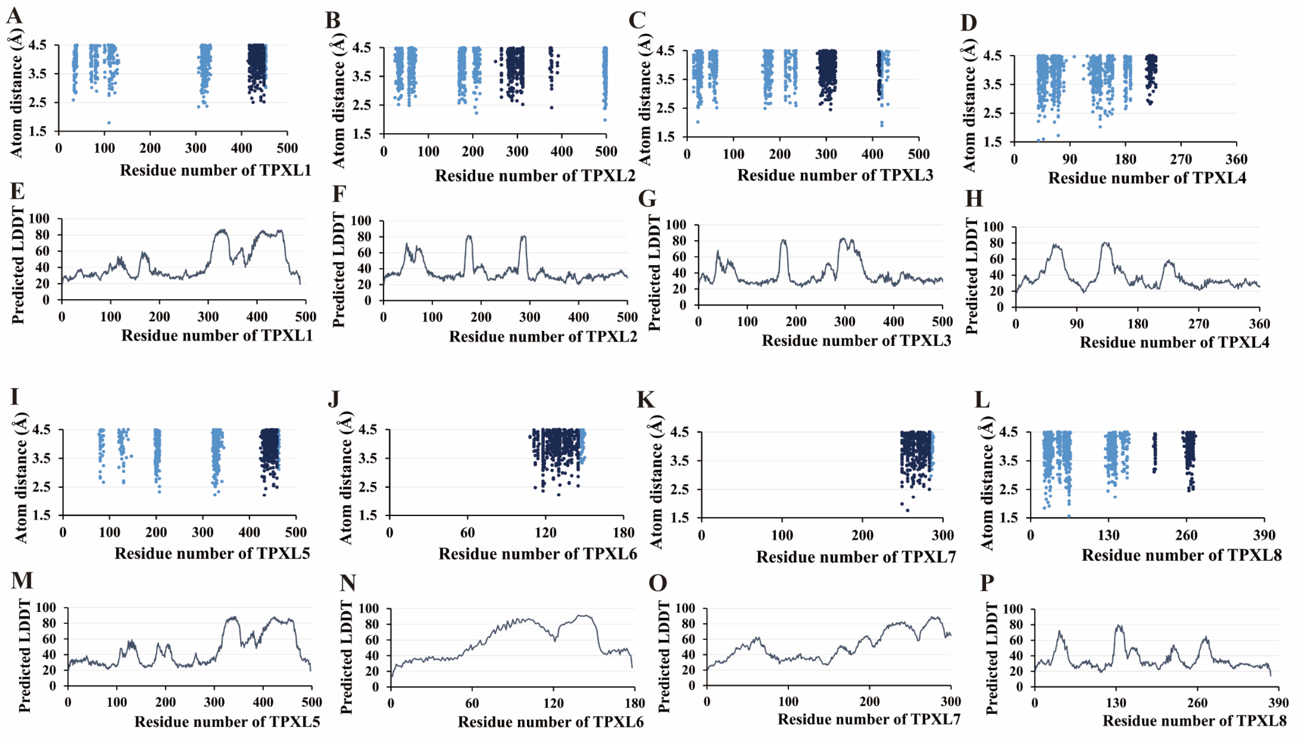

Figure S2. AlphaFold3 predicts that *Arabidopsis* TPXL proteins contact with tubulin. **(A–D and I–L)** Distribution of predicted atomic contacts between TPXL proteins and tubulin. The x-axis represents the amino acid positions of TPXL proteins, and the y-axis indicates the corresponding interatomic distances between TPXL and tubulin atoms. Contacts derived from non-TPX2 regions are shown as light blue dots, whereas contacts within the TPX2 domains are shown as dark blue dots. **(E–H and M–P)** Distribution of pLDDT scores across individual amino acid positions of TPXL proteins. Higher pLDDT values indicate greater confidence in the predicted local structure.

		TPXL1	TPXL2	TPXL3	TPXL4	TPXL5	TPXL6	TPXL7	TPXL8
Aurora 1	iPTM	0.14	0.77	0.8	0.79	0.12	0.19	0.16	0.77
	PTM	0.37	0.45	0.44	0.52	0.36	0.54	0.45	0.5
Aurora 2	iPTM	0.28	0.82	0.8	0.8	0.09	0.15	0.2	0.78
	PTM	0.37	0.46	0.45	0.52	0.36	0.55	0.46	0.49
Aurora 3	iPTM	0.25	0.77	0.76	0.72	0.3	0.2	0.14	0.75
	PTM	0.38	0.44	0.43	0.51	0.38	0.55	0.46	0.51

Figure S3. Confidence assessment of AlphaFold3-predicted TPXL–Aurora kinase complex models. Comparison of predicted interaction confidence scores for TPXL1–TPXL8 proteins in complex with *Arabidopsis* Aurora kinases AUR1, AUR2, and AUR3. The ipTM scores and pTM scores for each TPXL–Aurora kinase complex are shown.

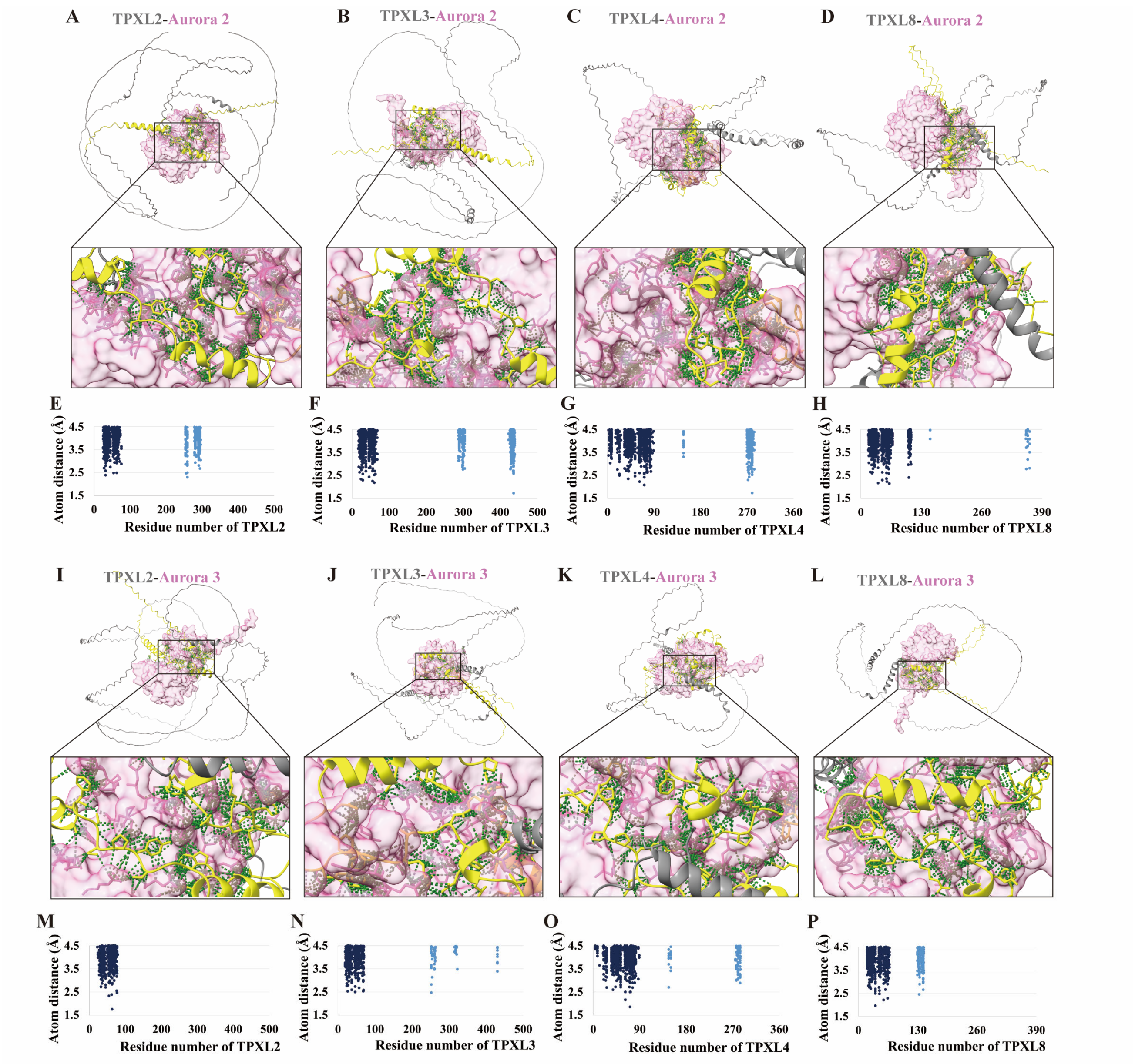

Figure S4. AlphaFold3 predicts Aurora-binding domain-mediated association between TPXL proteins and AUR1/AUR2. **(A–D and I–L)** Predicted structural models of TPXL2, TPXL3, TPXL4, and TPXL8 proteins in complex with *Arabidopsis* Aurora kinases (AUR2, and AUR3) generated using AlphaFold3. **(E–H and M–P)** Distribution of predicted atomic contacts between TPXL proteins and Aurora kinases.

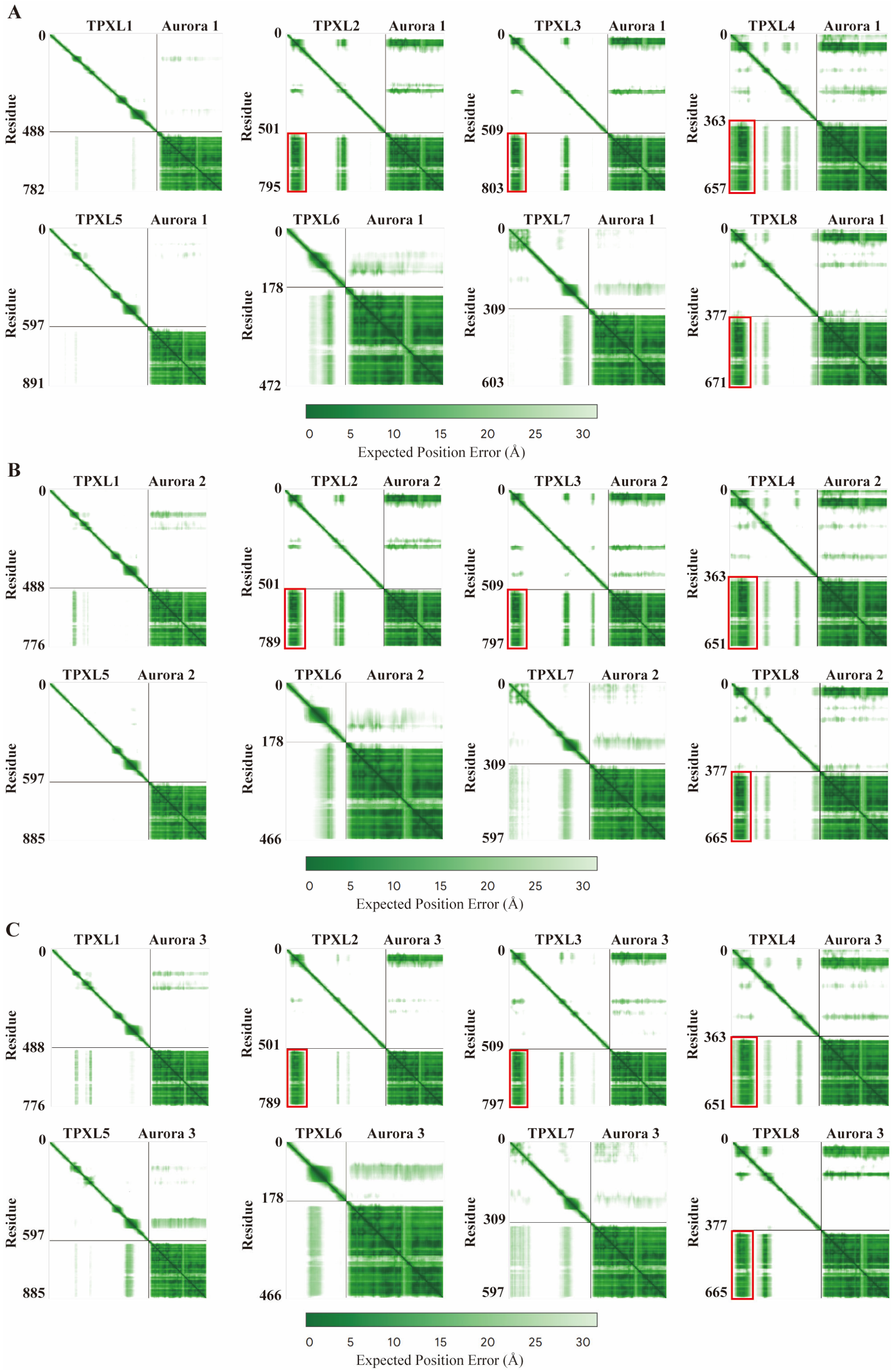

Figure S5. PAE analysis of AlphaFold3-predicted TPXL–Aurora kinase complex models. **(A–C)** PAE plots of TPXL1–TPXL8 proteins interacting with *Arabidopsis* Aurora kinases AUR1, AUR2, and AUR3, respectively. The PAE values indicate the predicted uncertainty between residue pairs within each TPXL–Aurora kinase complex. Lower PAE values at the predicted interaction interfaces indicate higher structural confidence of the corresponding regions.

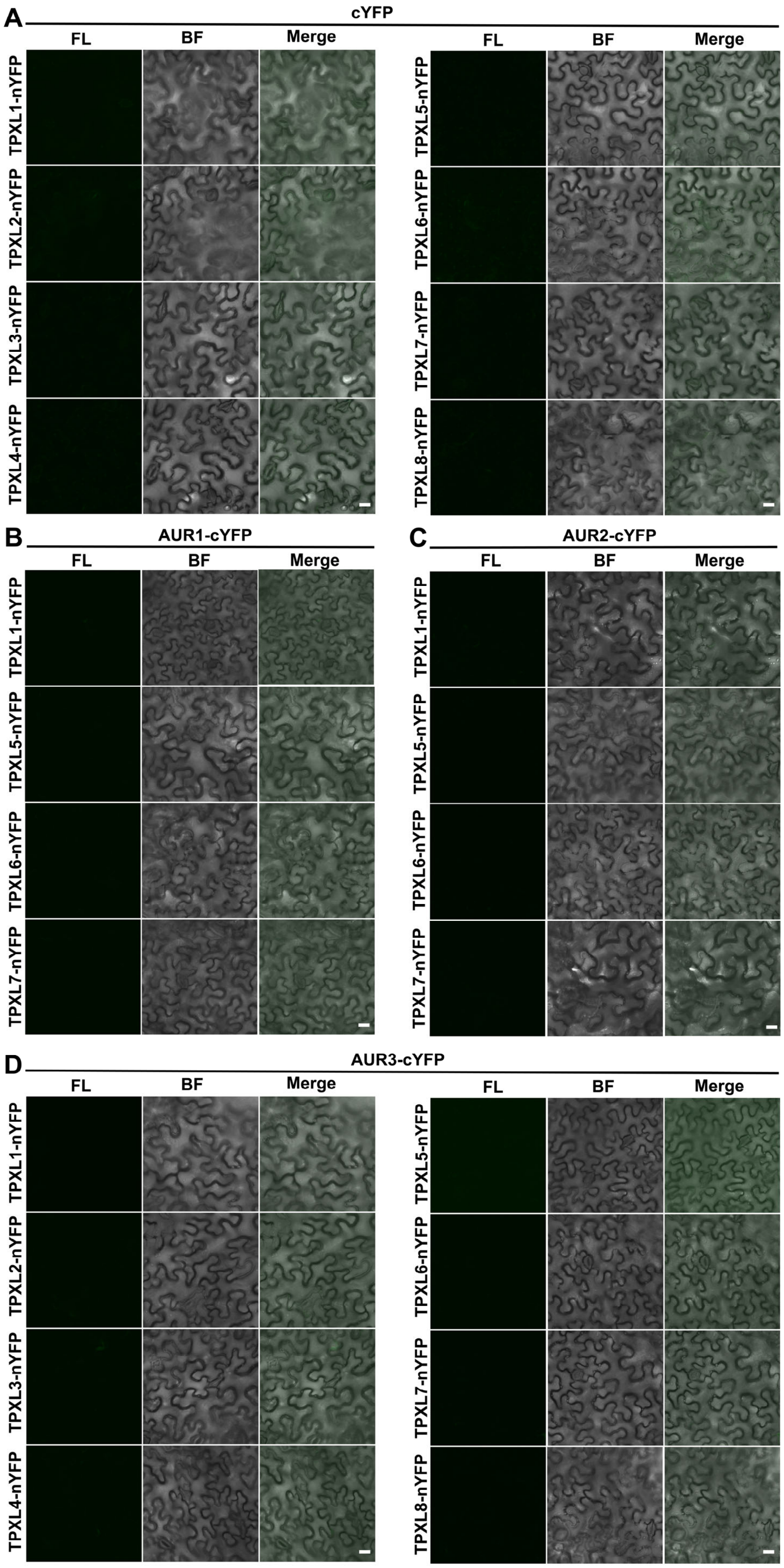
A
cYFP
FL
BF
Merge
TPXL1-nYFP
TPXL2-nYFP
TPXL3-nYFP
TPXL4-nYFP
TPXL5-nYFP
TPXL6-nYFP
TPXL7-nYFP
TPXL8-nYFP
B
AUR1-cYFP
TPXL1-nYFP
TPXL5-nYFP
TPXL6-nYFP
TPXL7-nYFP
C
AUR2-cYFP
TPXL1-nYFP
TPXL5-nYFP
TPXL6-nYFP
TPXL7-nYFP
D
AUR3-cYFP
TPXL1-nYFP
TPXL2-nYFP
TPXL3-nYFP
TPXL4-nYFP
TPXL5-nYFP
TPXL6-nYFP
TPXL7-nYFP
TPXL8-nYFP

Figure S6. BiFC analysis of TPXL proteins showing no detectable interaction with Aurora kinases. **(A)** These negative controls were performed in *Nicotiana benthamiana* leaf epidermal cells in BiFC assays. No reconstituted YFP fluorescence was detected. **(B-D)** BiFC assays were performed in *Nicotiana benthamiana* leaf epidermal cells to examine interactions between TPXL proteins and Aurora kinases. No reconstituted YFP fluorescence was detected for the indicated TPXL–Aurora combinations, suggesting the absence of detectable interaction under the tested conditions. FL: Fluorescence, BF: Brightfield. All experiments were repeated at least three times, and at least 3 cells were analyzed each time. Scale bars, 20 μm.

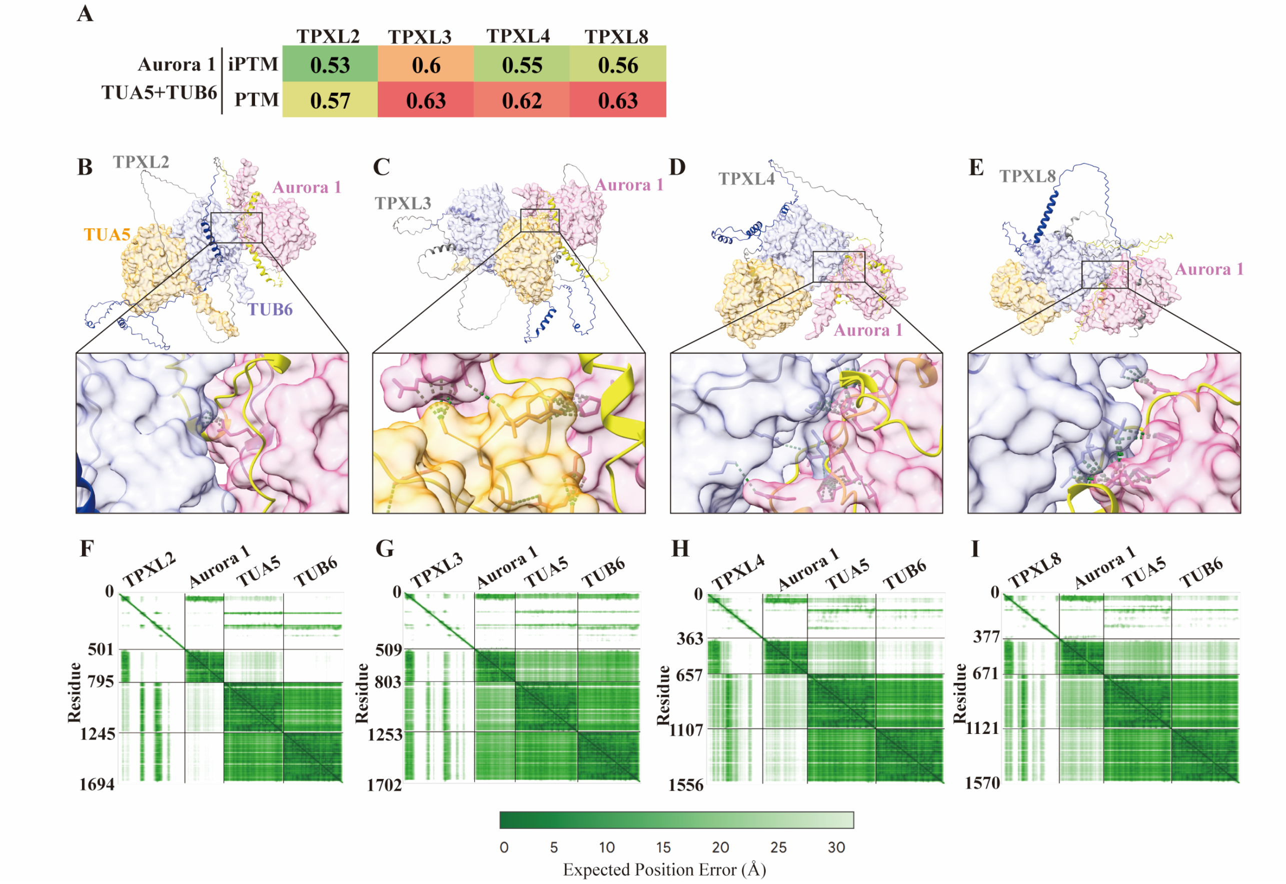

Figure S7. AlphaFold3 predicts TPXL-mediated association between Aurora kinase and tubulin. **(A)** Prediction confidence evaluation of TPXL2, TPXL3, TPXL4, and TPXL8 ternary complexes with AUR1 and the tubulin heterodimer TUA5/TUB6. The ipTM and pTM scores of the predicted complexes are shown. **(B–E)** Predicted structural models of TPXL2, TPXL3, TPXL4, and TPXL8 in complex with AUR1 and the TUA5/TUB6 tubulin heterodimer generated using AlphaFold3. TPXL proteins are shown in gray, TPX2 domains are highlighted in blue, Aurora-binding domains are highlighted in yellow, AUR1 is shown in pink, and tubulin subunits are shown in orange and purple. The predicted atomic distances between AUR1 and tubulin atoms are indicated by green dashed lines. Few AUR1–tubulin atom pairs were identified within the 4.5 Å cutoff, suggesting limited direct contact between Aurora kinase and tubulin. **(F–I)** PAE plots of TPXL2, TPXL3, TPXL4, and TPXL8 ternary complexes with AUR1 and TUA5/TUB6. Lower PAE values indicate higher confidence in the predicted relative positioning of residues within the modeled complexes.

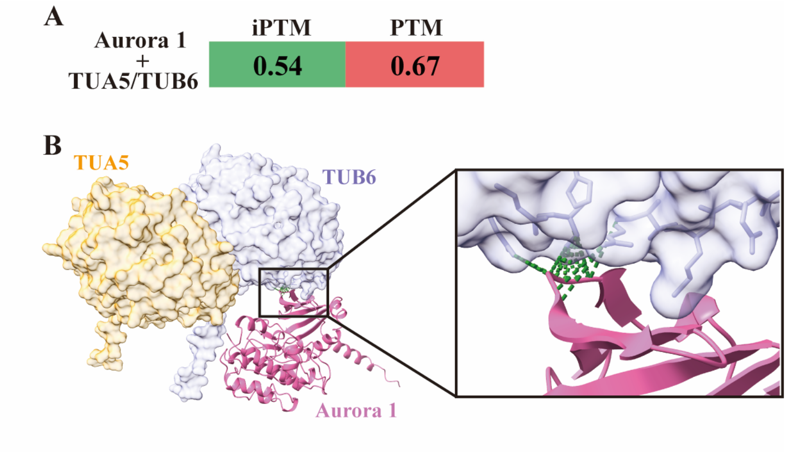

Figure S8. AlphaFold3 prediction of AUR1 association with tubulin. **(A)** Confidence metrics of predicted AUR1–TUA5/TUB6 ternary complexes, including ipTM and pTM values. **(B)** Predicted structural models of AUR1 in complex with the TUA5/TUB6 tubulin heterodimer generated using AlphaFold3. AUR1 in pink, and tubulin subunits TUA5 and TUB6 in orange-yellow/purple, respectively. Predicted atomic contacts between different protein components within 4.5 Å are indicated by green dashed lines.

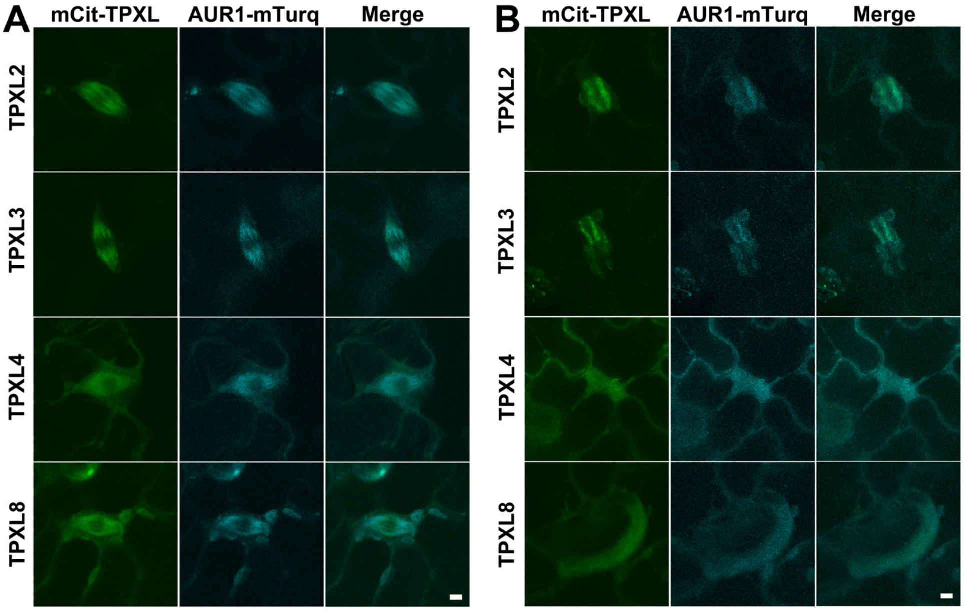

Figure S9. Aurora1 was transiently co-expressed with several TPXL proteins during cell division. **(A-B)** MCitrine-TPXL2, TPXL3, TPXL4, and TPXL8 (green) transiently co-expressed with AUR1-mTurquoise2 (cyan) during cell division in *Nicotiana benthamiana* leaf epidermal cells. Mitotic cells were induced by co-expression of AtCYCD3;1. During metaphase, AUR1 localized along the spindle microtubules. During cytokinesis, AUR1 accumulated at the phragmoplast microtubule array, showing localization patterns similar to TPXL proteins. All experiments were repeated three times, and at least 3 cells were analyzed each time. Scale bars, 5 µm.
