# Supplementary material for: Arabidopsis TPXL Proteins Mediate a Selective Aurora Kinase–Microtubule Association During Cell Division
Source: Int J Mol Sci. 2026 Sep 8;27(18):7980. doi: 10.3390/ijms27187980 (PMC13606213; doi:10.3390/ijms27187980)
Supplement: Supplementary file 1 [file ijms-27-07980-s001.zip › Supplementary Table-TPXL-Revised manuscript-dpz.pdf]

Table S1. Protein sequences used for AlphaFold3 prediction

Protein name	TAIR accession	Protein length (aa)
TPXL1	AT3G01015.1	488
TPXL2	AT4G11990.1	501
TPXL3	AT4G22860.1	509
TPXL4	AT5G07170.1	363
TPXL5	AT5G15510.1	497
TPXL6	AT5G37478.1	178
TPXL7	AT5G44270.1	309
TPXL8	AT5G62240.1	377
AUR 1	AT4G32830.1	294
AUR 2	AT2G25880.1	288
AUR 3	AT2G45490.1	288
TUA2	AT1G50010.1	450
TUA5	AT5G19780.1	450
TUB4	AT5G44340.1	444
TUB6	AT5G12250.1	449

Table S2. Amino acid coordinates of TPX2 and Aurora-binding domains used for structural analyses

Protein name	Domain	Amino acid coordinates (aa)	Annotation source
TPXL1	TPX2 domain	375 - 449	TAIR
TPXL2	TPX2 domain	244-408	TAIR
TPXL3	TPX2 domain	253-415	TAIR
TPXL4	TPX2 domain	210-317	TAIR
TPXL5	TPX2 domain	385-459	TAIR
TPXL6	TPX2 domain	70-145	TAIR
TPXL7	TPX2 domain	208-283	TAIR
TPXL8	TPX2 domain	198-331	TAIR
TPXL1	Aurora-binding domain	/	not annotated
TPXL2	Aurora-binding domain	1-100	Previous annotation
TPXL3	Aurora-binding domain	1-100	Previous annotation
TPXL4	Aurora-binding domain	1-100	Previous annotation
TPXL5	Aurora-binding domain	/	not annotated
TPXL6	Aurora-binding domain	/	not annotated
TPXL7	Aurora-binding domain	/	not annotated
TPXL8	Aurora-binding domain	1-100	Previous annotation

Table S3. List of primers used for plasmid construction in this study

Primer name	Sequence (5′–3′)	Purpose/ Vector
pUBQ14-F	CTATGGATCCGGCCTCGAGATCCGAACAGAGTTAAACCGG	Fluorescent fusion construct/ *pEG101-pUBQ14-mCitrine-TPXL*
pUBQ14-R	TGATGATTTTCCAGTACACATCCCGGGAAACTGAGATTAATCGCTTGGATAATCT	
mCitrine-F	AGAGGTGGTAGTGGTGGTAGTATGGTGAGCAAGGGCG	Fluorescent fusion construct/ *pEG101-pUBQ14-mCitrine-TPXL*
mCitrine-R	GACTCTAGGGACTAGTCTACAGCTTGGATCCGGAC	
TPXL1-F	ATCCAAGCTGTCTAGAGGTGGTAGTGGTGGTAGTATGGAGTCGACGAATTTGAAG	Fluorescent fusion construct/ *pEG101-pUBQ14-mCitrine-TPXL1*
TPXL1-R	GACTCTAGGGACTAGTTTAGAGGTCTTGTTGGTATAAGAGGT	
TPXL2-F	ATCCAAGCTGTCTAGAGGTGGTAGTGGTGGTAGTATGGAGATGGAGACGGAC	Fluorescent fusion construct/ *pEG101-pUBQ14-mCitrine-TPXL2*
TPXL2-R	GACTCTAGGGACTAGTTTAATTTAGTTGCACCGTTGCT	
TPXL3-F	ATCCAAGCTGTCTAGAGGTGGTAGTGGTGGTAGTATGGAGATGGATGAAGATATGGA	Fluorescent fusion construct/ *pEG101-pUBQ14-mCitrine-TPXL3*
TPXL3-R	GACTCTAGGGACTAGTTCAGCGAATGCCTAAGTTCC	
TPXL4-F	ATCCAAGCTGTCTAGAGGTGGTAGTGGTGGTAGTATGCTCGATTTCAACATGTTCT	Fluorescent fusion construct/ *pEG101-pUBQ14-mCitrine-TPXL4*
TPXL4-R	GACTCTAGGGACTAGTTCAAATTCCTTCAATAAACCTCAGAAG	

TPXL5-F	GAGGTGGTAGTGGTGGTAGTATGG AGTCGTTGACATTGAAG	Fluorescent fusion construct/ *pEG101-pUBQ14-mCitrine-TPXL5*
TPXL5-R	GCAGGACTCTAGGGACTAGTCTAGA GAAACTGATATTGGAAATCGC	
TPXL6-F	AGGTGGTAGTGGTGGTAGTATGTGT ACTGGAAAATCATCATACAC	Fluorescent fusion construct/ *pEG101-pUBQ14-mCitrine-TPXL6*
TPXL6-R	GCAGGACTCTAGGGACTAGTTTAAG CATGAGCATGGTTAATATGG	
TPXL7-F	ATCCAAGCTGTCTAGAGGTGGTAGT GGTGGTAGTATGGAAACAAAAATC ACATTTGTTT	Fluorescent fusion construct/ *pEG101-pUBQ14-mCitrine-TPXL7*
TPXL7-R	GACTCTAGGGACTAGTCTACCTCAT GTGAGGAGAAAC	
TPXL8-F	ATCCAAGCTGTCTAGAGGTGGTAGT GGTGGTAGTATGGTTCCCGAATCAT CATC	Fluorescent fusion construct/ *pEG101-pUBQ14-mCitrine-TPXL8*
TPXL8-R	GACTCTAGGGACTAGTTTAAGCAGT GATTAGGTTCCTTG	
TUA5-F	TAGTGGTGGTAGTGGTGGTAGTATG AGGGAAATTATTAGCATTCATATCG	Fluorescent fusion construct/ *pGWB2-pUBQ14-tdTomoto-TUA5*
TUA5-R	CGATCGGGGAAATTCGAGCTCTCAA TAGTCTTCACCTTCATCTTCTTC	
pGWB2-pUBQ14-F	ACCATGATTACGCCAAGCTTATCCG AACAGAGTTAAACCGGG	Fluorescent fusion construct/ *pGWB2-pUBQ14-tdTomoto-TUA5*
pGWB2-pUBQ14-R	AACTTGTTGATAACTCTAGAAAACT GAGATTAATCGCTTGGA	
tdTomato-F	GAGAACACGGGGGACTCTAGAATG GTGAGCAAGGGCGAGG	Fluorescent fusion construct/ *pGWB2-pUBQ14-tdTomoto-TUA5*
tdTomato-R	TACCACCACTACCACCACTAGTCTT GTACAGCTCGTCCATGCC	
mTurquoise2-F	ACTAGTGGTACCATGGTGAGCAAG GGCGA	Fluorescent fusion construct/ *pCAMBIA1300-pRPS5A-AUR1-mTurquoise2*
mTurquoise2-R	CGGGGAAATTCGAGCTCTTACTTGT ACAGCTCGTCCAT	
AUR1-F	TTCTCTCACCACAGCCATGGCGATC CCTACGG	Fluorescent fusion construct/ *pCAMBIA1300-pRPS5A-AUR1-*

AUR1-R	CCATGGTACCACTAGTAACTCTGTAGATTCCAGAAGGAT	*mTurquoise2*
AD-TPXL1-F	TGGCCATGGAGGCCGAATTCATGGAGTCGACGAATTTGAAGA	Yeast two-hybrid AD-TPXL1 construct
AD-TPXL1-R	GCGGCCGCTGCAGGTCGACTTAGAGGTCTTGTTGGTATAAGAGG	
AD-TPXL2-F	GGAGGCCAGTGAATTCATGGAGATGGAGACGGAC	Yeast two-hybrid AD-TPXL2 construct
AD-TPXL2-R	CGAGCTCGATGGATCCTTAATTTAGTTGCACCGTTGCT	
AD-TPXL3-F	GGAGGCCAGTGAATTCATGGAGATGGATGAAGATATGGAA	Yeast two-hybrid AD-TPXL3 construct
AD-TPXL3-R	CGAGCTCGATGGATCCTCAGCGAATGCCTAAGTTCC	
AD-TPXL4-F	GGAGGCCAGTGAATTCATGCTCGATTTCAACATGTTCT	Yeast two-hybrid AD-TPXL4 construct
AD-TPXL4-R	CGAGCTCGATGGATCCTCAAATTCCTTCAATAAACCTCAGAAG	
AD-TPXL5-F	CCATGGAGGCCAGTGAATTCATGGAGTCGTTGACATTGAAGA	Yeast two-hybrid AD-TPXL5 construct
AD-TPXL5-R	AGCTCGAGCTCGATGGATCCCTAGAGAAACTGATATTGGAAATCGC	
AD-TPXL6-F	CCATGGAGGCCAGTGAATTCATGTGTACTGGAAAATCATCATACACA	Yeast two-hybrid AD-TPXL6 construct
AD-TPXL6-R	AGCTCGAGCTCGATGGATCCTTAAGCATGAGCATGGTTAATATGGTA	
AD-TPXL7-F	GGAGGCCAGTGAATTCATGGAAACAAAAATCACATTTGTTTGG	Yeast two-hybrid AD-TPXL7 construct
AD-TPXL7-R	CGAGCTCGATGGATCCCTACCTCATGTGAGGAGAAACC	
AD-TPXL8-F	GGAGGCCAGTGAATTCATGGTTCCCGAATCATCATCG	Yeast two-hybrid AD-TPXL8 construct
AD-TPXL8-R	CGAGCTCGATGGATCCTTAAGCAGTGATTAGGTTCCTTGC	
BD-Aurora 1-F	TGGCCATGGAGGCCGAATTCATGGCGATCCCTACGGAG	Yeast two-hybrid

BD-Aurora 1-R	TGCGGCCGCTGCAGGTCGACTTAAA CTCTGTAGATTCCAGAAGGATC	BD-Aurora 1 construct
BD-Aurora 2-F	TGGCCATGGAGGCCGAATTCATGGG GATTTCTACAGAGACGC	Yeast two-hybrid
BD-Aurora 2-R	TGCGGCCGCTGCAGGTCGACTCATC CTCTGTAAAGGCCTGA	BD-Aurora 2 construct
BD-Aurora 3-F	TGGCCATGGAGGCCGAATTCATGAG TAAGAAATCGACAGAATCTGAC	Yeast two-hybrid
BD-Aurora 3-R	TGCGGCCGCTGCAGGTCGACTCAAA TATCAATTGAGGCACACAC	BD-Aurora 3 construct
TPXL1-nYFP-F	CACGGGGGACTCTAGAATGGAGTC GACGAATTTGAAG	BiFC fusion construct/
TPXL1-nYFP-R	TAGTGGATCCCCCGGGGAGGTCTTG TTGGTATAAGAGGTC	*pCAMBIA1300-TPXL1-nYFP*
TPXL2-nYFP-F	CACGGGGGACTCTAGAATGGAGAT GGAGACGGACA	BiFC fusion construct/
TPXL2-nYFP-R	TAGTGGATCCCCCGGGATTTAGTTG CACCGTTGCTTC	*pCAMBIA1300-TPXL2-nYFP*
TPXL3-nYFP-F	CACGGGGGACTCTAGAATGGAGAT GGATGAAGATATGGAAA	BiFC fusion construct/
TPXL3-nYFP-R	TAGTGGATCCCCCGGGGCGAATGCC TAAGTTCCTC	*pCAMBIA1300-TPXL3-nYFP*
TPXL4-nYFP-F	CACGGGGGACTCTAGAATGCTCGAT TTCAACATGTTCT	BiFC fusion construct/
TPXL4-nYFP-R	TAGTGGATCCCCCGGGAATTCCTTC AATAAACTAAAAACCTGGT	*pCAMBIA1300-TPXL4-nYFP*
TPXL5-nYFP-F	GAACACGGGGGACTCTAGAATGGA GTCGTTGACATTGAAG	BiFC fusion construct/
TPXL5-nYFP-R	GCGGTACCCTCGAGGTCGACGAGA AACTGATATTGGAAATCGC	*pCAMBIA1300-TPXL5-nYFP*
TPXL6-nYFP-F	GAACACGGGGGACTCTAGAATGTG TACTGGAAAATCATCATACACA	BiFC fusion construct/
TPXL6-nYFP-R	GCGGTACCCTCGAGGTCGACAGCAT GAGCATGGTTAATATGGTA	*pCAMBIA1300-TPXL6-nYFP*
TPXL7-nYFP-F	CACGGGGGACTCTAGAATGGAAAC AAAAATCACATTTGTTTG	BiFC fusion construct/

TPXL7-nYFP-R	TAGTGGATCCCCCGGGCCTCATGTGAGGAGAAACCA	*pCAMBIA1300-TPXL7-nYFP*
TPXL8-nYFP-F	CACGGGGGACTCTAGAATGGTTCCCGAATCATCATC	BiFC fusion construct/ *pCAMBIA1300-TPXL8-nYFP*
TPXL8-nYFP-R	TAGTGGATCCCCCGGGAGCAGTGATTAGGTTCCTTGC	
AUR1-cYFP-F	GGCGCGCCACTAGTGGATCCATGGCGATCCCTACGGAG	BiFC fusion construct/ *pCAMBIA1300-Aurora 1-cYFP*
AUR1-cYFP-R	GCGGTACCCTCGAGGTCGACTTAAACTCTGTAGATTCCAGAAGGATC	
AUR2-cYFP-F	GGCGCGCCACTAGTGGATCCATGGGGATTTCTACAGAGACGC	BiFC fusion construct/ *pCAMBIA1300-Aurora 2-cYFP*
AUR2-cYFP-R	GCGGTACCCTCGAGGTCGACTCATCCTCTGTAAAGGCCTGA	
AUR3-cYFP-F	GGCGCGCCACTAGTGGATCCATGAGTAAGAAATCGACAGAATCTGAC	BiFC fusion construct/ *pCAMBIA1300-Aurora 3-cYFP*
AUR3-cYFP-R	GCGGTACCCTCGAGGTCGACTCAAATATCAATTGAGGCACACAC	
